# Supplementary material for: Identification of spatially variable genes with graph cuts
Source: Nat Commun. 2022 Sep 19;13:5488. doi: 10.1038/s41467-022-33182-3 (PMC9485129; doi:10.1038/s41467-022-33182-3)
Supplement: Supplementary file 1 — Supplementary Information [file 41467_2022_33182_MOESM1_ESM.pdf]

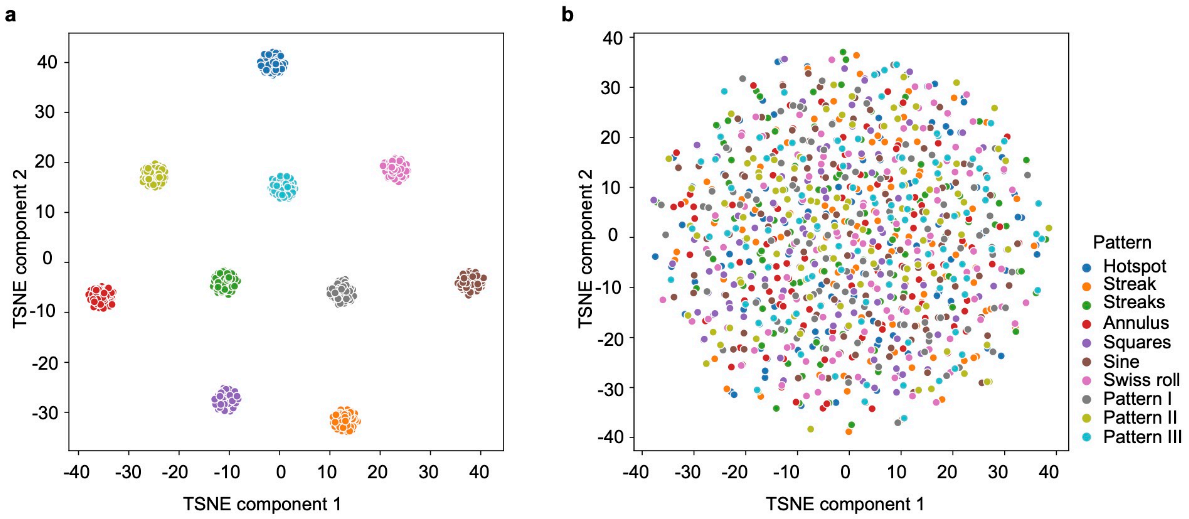

Supplementary Figure 1. T-SNE analysis of simulated data.

(a) t-SNE projections showing that the simulated SV genes formed 10 well-separated clusters. **(b)** t-SNE projections showing that no clusters were formed among random genes whose gene expression values were randomly shuffled.

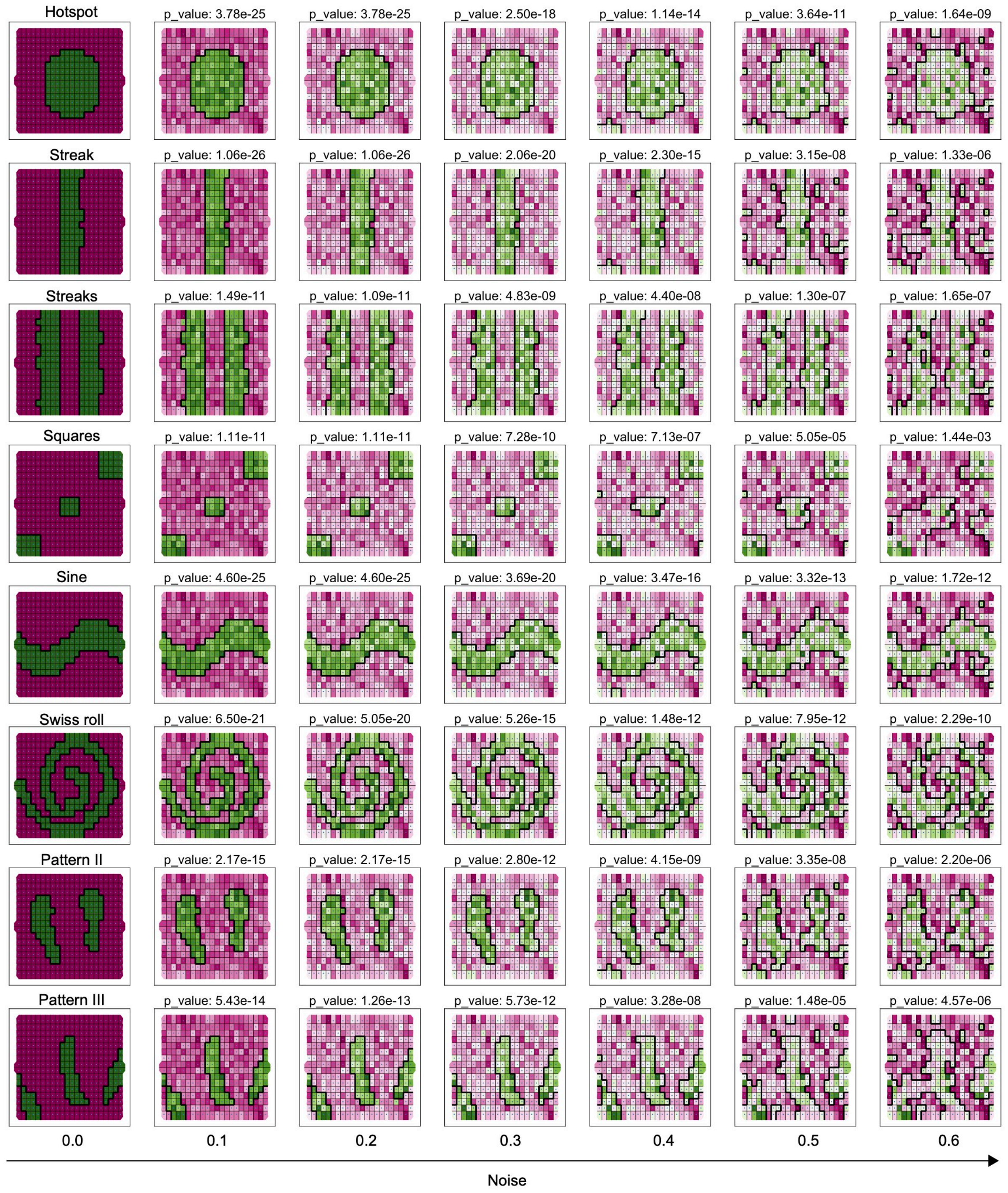

Supplementary Figure 2. Performance of SV gene identification algorithms using simulated data with Gaussian noises.

Representative graph cuts results at increasing Gaussian noise levels for scGCO. P-values were calculated by scGCO without multiple-testing correction. Turquoise denotes high expression and magenta denotes low expression.

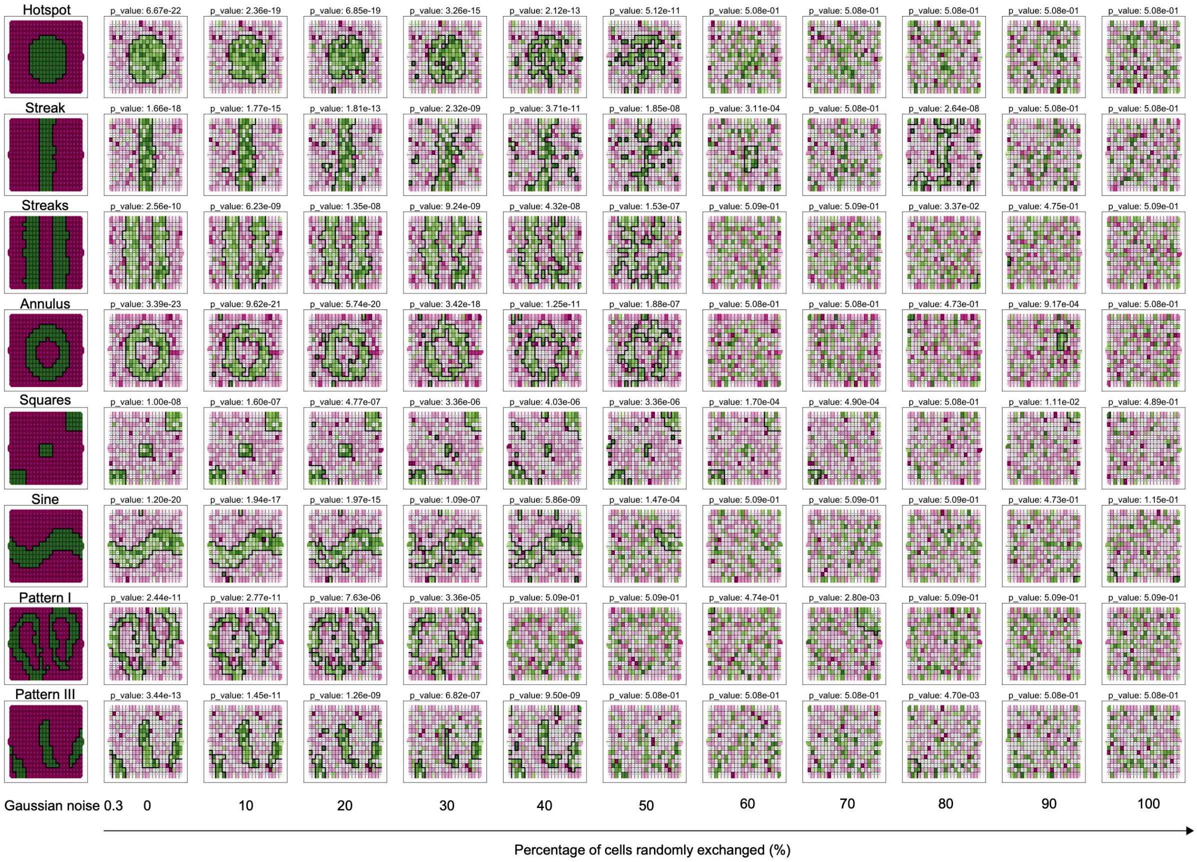

Supplementary Figure 3. Performance of SV gene identification algorithms using simulated data with random exchanges.

Representative graph cuts results at increasing percentages of randomly exchanged cells for scGCO. P-values were calculated by scGCO without multiple-testing correction. Turquoise denotes high expression and magenta denotes low expression.

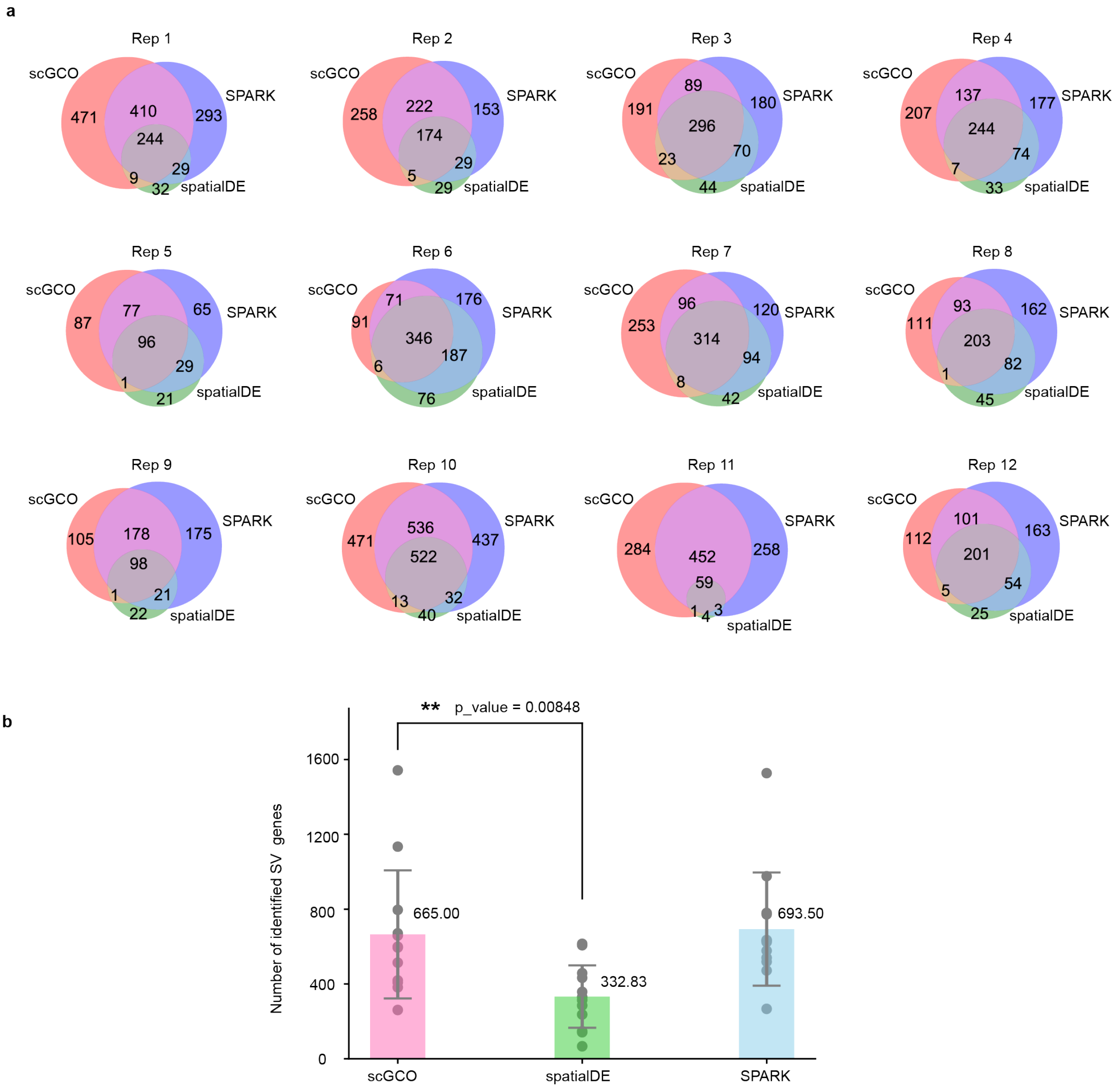

Supplementary Figure 4. ScGCO identifies SV genes from mouse olfactory bulb data.

(**a**) Venn diagrams showing the set relationship among SV genes identified by scGCO (FDR <0.05), spatialDE (FDR <0.05), and SPARK (FDR <0.05) in mouse olfactory bulb data. **(b)** Bar plots showing the number of identified SV genes from mouse olfactory bulb dataset (n=12 replicates) by different methods. Error bars indicate means ± SD. The p-value ($p = 0.00848$, scGCO vs. spatialDE) was determined using the two-sided pairwise t-test without multiple-testing correction.

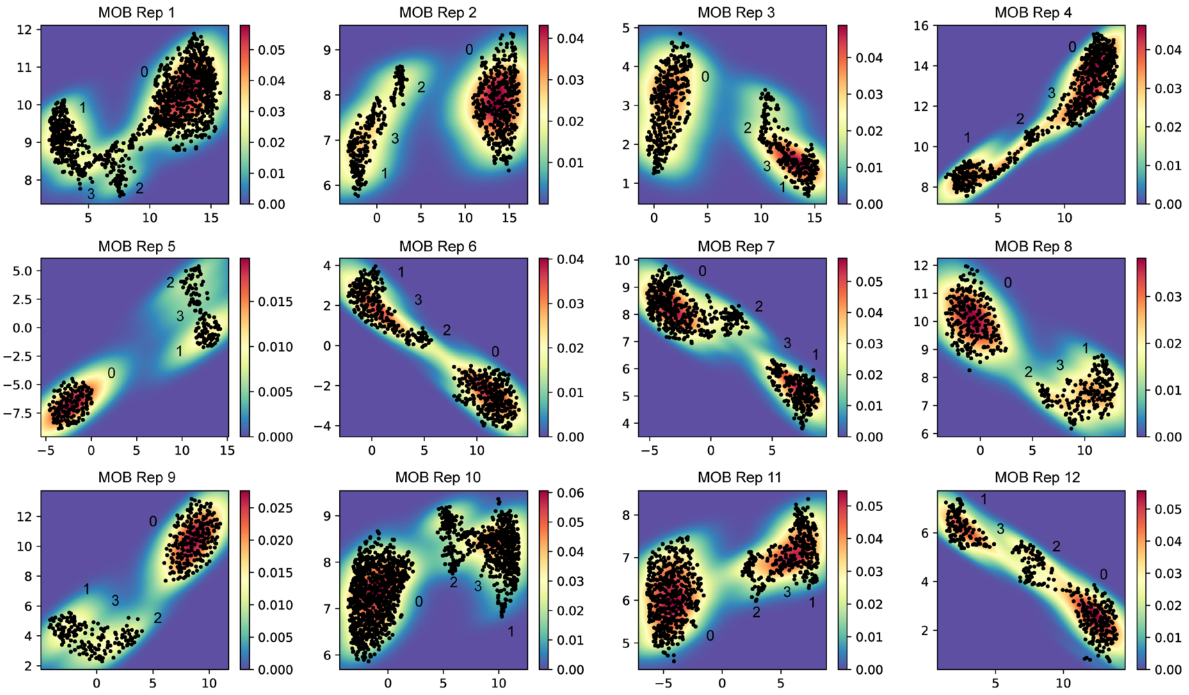

Supplementary Figure 5. Clustering analyses of SV genes identified by scGCO in mouse olfactory bulb data.

UMAP analyses of SV genes identified by scGCO for all 12 replicates of mouse olfactory bulb data. Each point is a gene. Background color indicates density of points determined with kernel density estimation. Numbers indicate identified clusters by HDBSCAN.

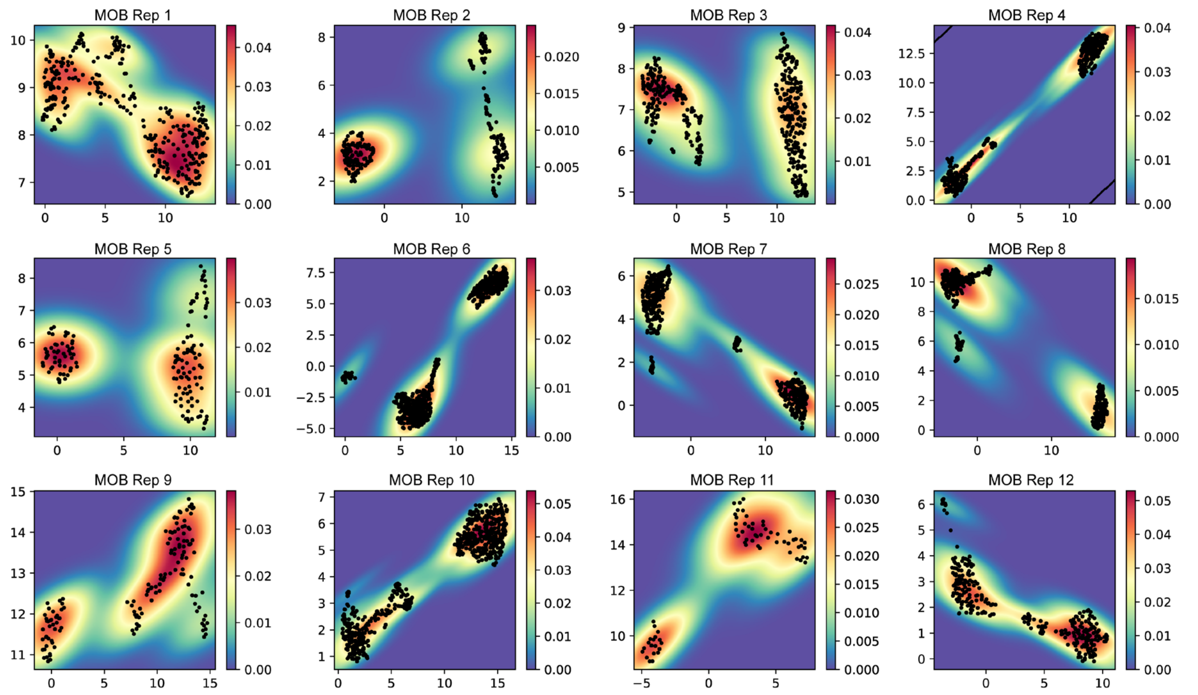

Supplementary Figure 6. Clustering analyses of SV genes identified by spatialDE in mouse olfactory bulb data.

UMAP analyses of SV genes identified by spatialDE for all 12 replicates of mouse olfactory bulb data. Each point is a gene. Background color indicates density of points determined by kernel density estimation.

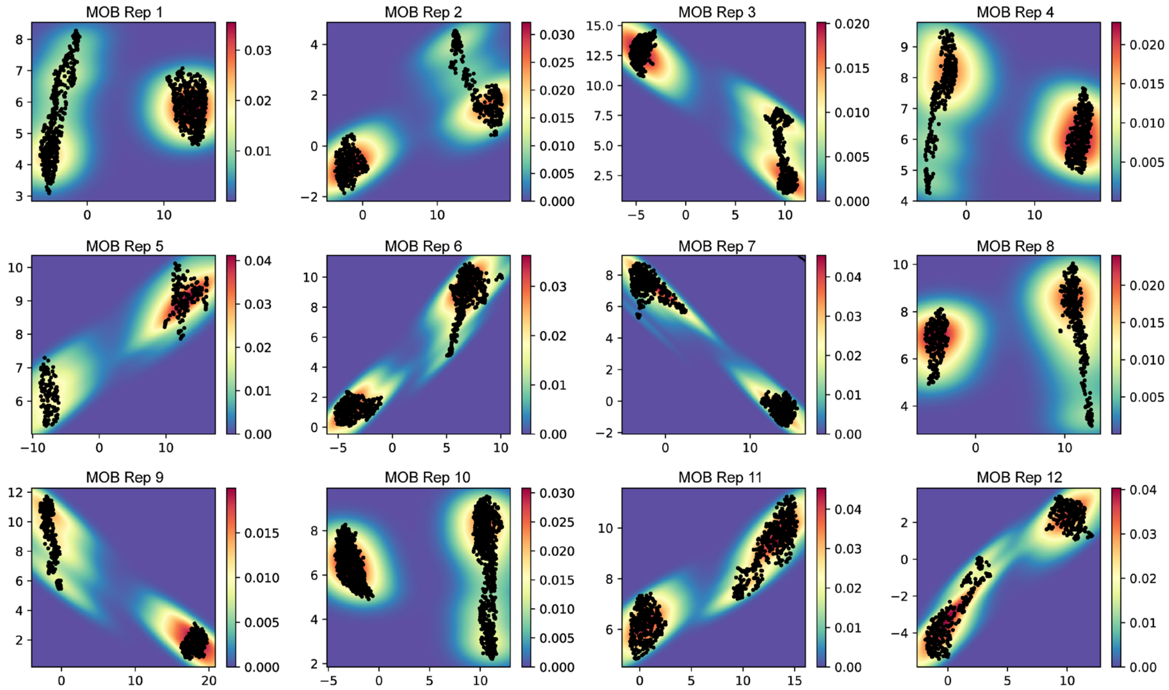

Supplementary Figure 7. Clustering analyses of SV genes identified by SPARK in mouse olfactory bulb data.

UMAP analyses of SV genes identified by SPARK for all 12 replicates of mouse olfactory bulb data. Each point is a gene. Background color indicates density of points determined by kernel density estimation.

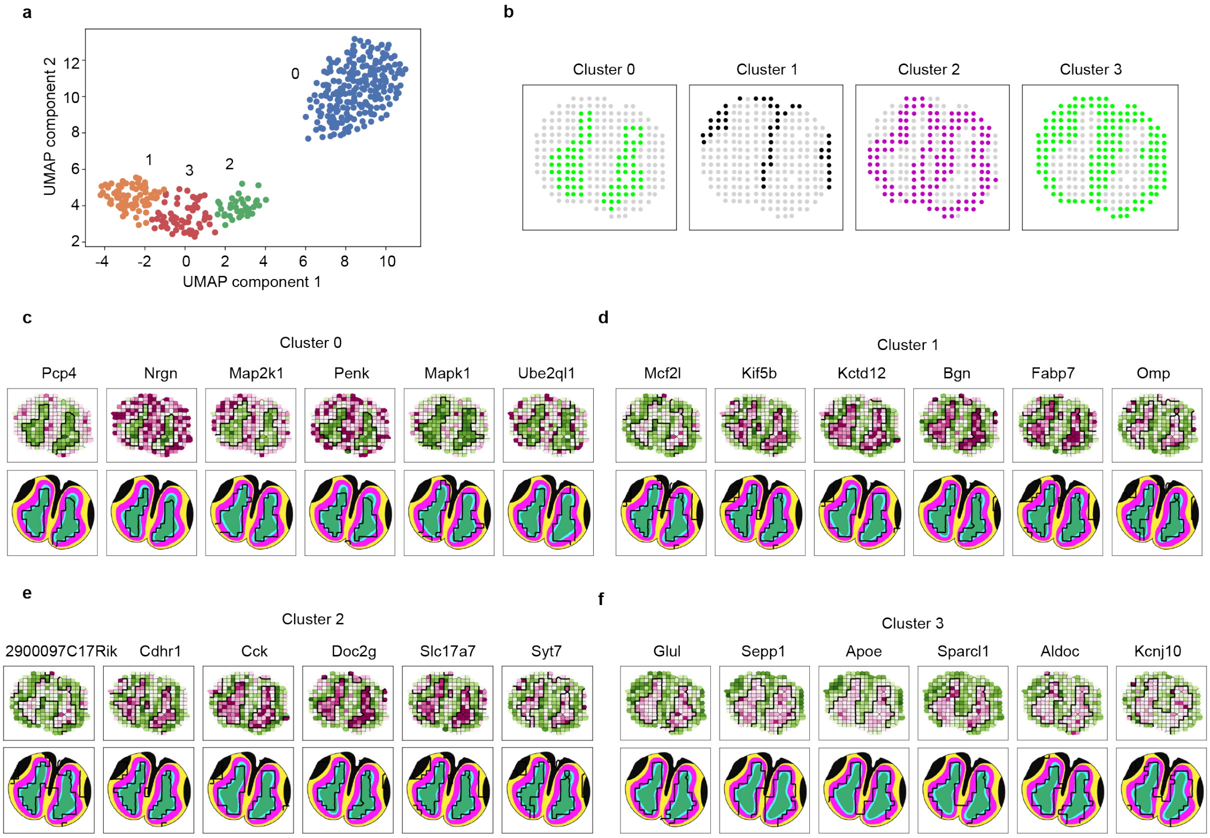

Supplementary Figure 8. Identification of SV genes in replicate 9 of MOB data by scGCO.

(**a**) UMAP for SV genes identified by scGCO in replicate 9 of MOB data. **(b)** Tissue structures resolved with each cluster of SV genes identified by scGCO for replicate 9. **(c)** Representative Voronoi diagrams showing graph cuts results (top panels), and associated tissue structure overlays (lower panels) for cluster 0. **(d-f)** Same as **(c)** for cluster 1, 2 and 3 respectively. Six examples were shown for each cluster. Turquoise denotes high expression and magenta denotes low expression. Thicker black lines denote graph cuts boundaries.

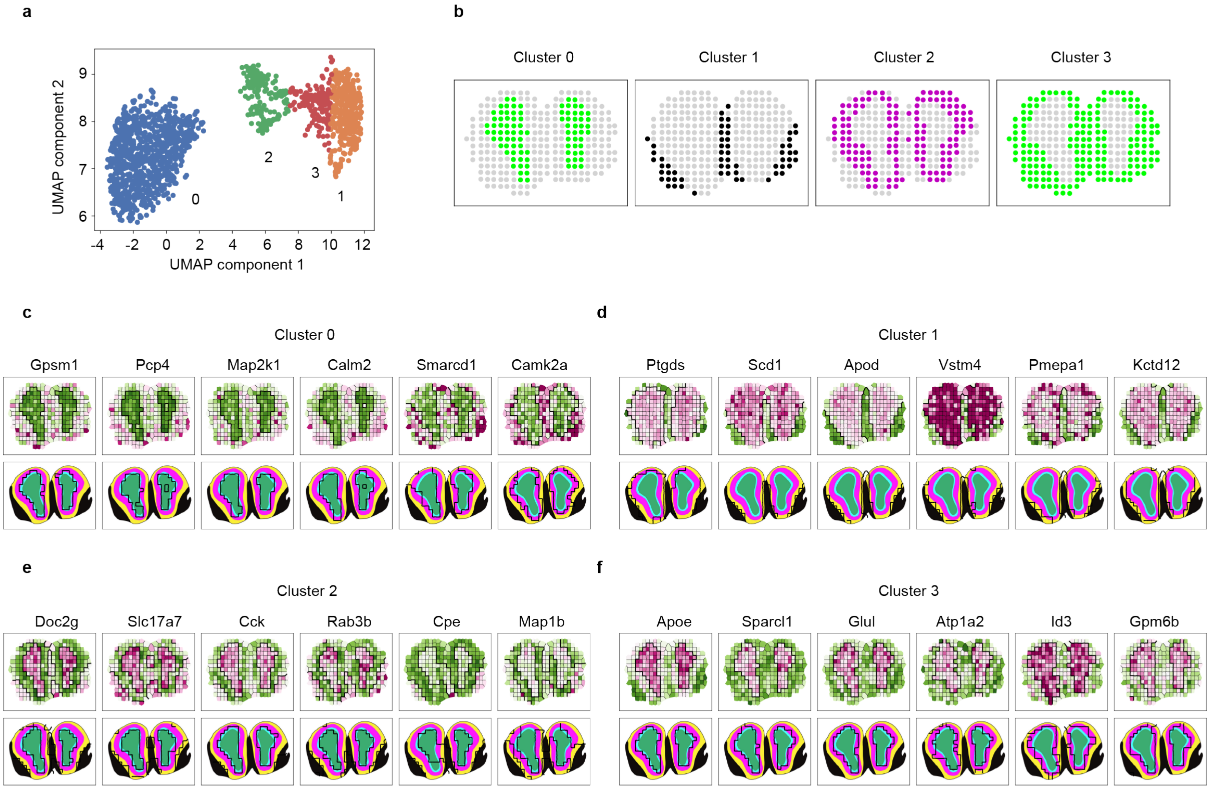

Supplementary Figure 9. Identification of SV genes in replicate 10 of MOB data by scGCO.

(**a**) UMAP for SV genes identified by scGCO in replicate 10 of MOB data. **(b)** Tissue structures resolved with each cluster of SV genes identified by scGCO for replicate 10. **(c)** Representative Voronoi diagrams showing graph cuts results (top panels), and associated tissue structure overlays (lower panels) for cluster 0. **(d-f)** Same as **(c)** for cluster 1, 2 and 3 respectively. Six examples were shown for each cluster. Turquoise denotes high expression and magenta denotes low expression. Thicker black lines denote graph cuts boundaries.

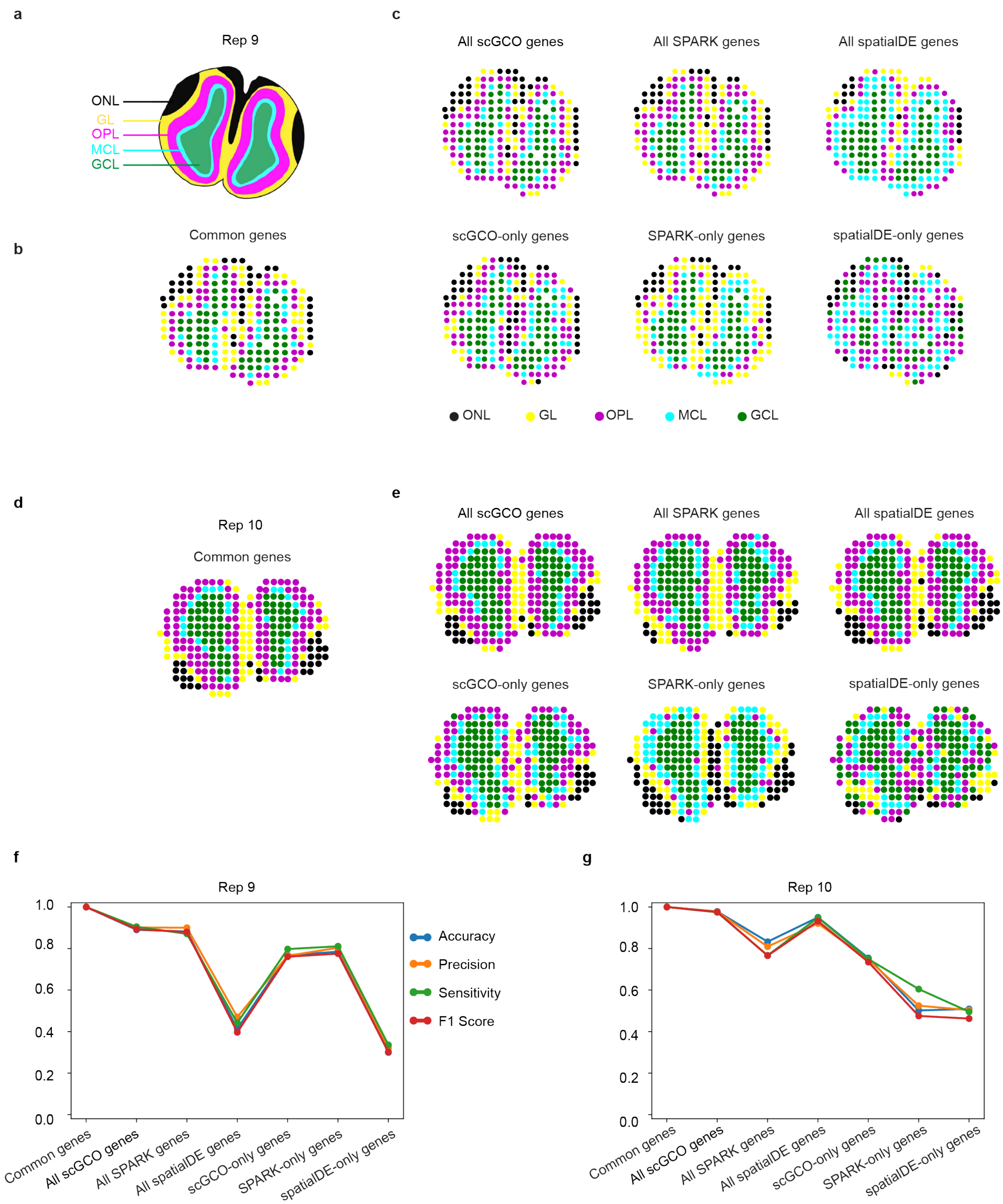

Supplementary Figure 10. SV genes uniquely identified by scGCO resolved MOB tissue structures.

(**a**) Annotation of the MOB tissue architecture showing the five known layers for replicate 9. ONL: olfactory nerve layer; GL: glomerular layer; OPL: outer plexiform layer; MCL: mitral cell layer; GCL: granular cell layer. **(b)** MOB tissue architecture reconstructed using SV genes jointly identified by the three methods (scGCO, spatialDE and SPARK) for replicate 9.

(c) The MOB tissue structures reconstructed using all scGCO genes (upper column 1), all SPARK genes (upper column 2), all spatialDE genes (upper column 3), scGCO-only genes (lower column 1), SPARK-only genes (lower column 2), and spatialDE-only genes (lower column 3) for replicate 9. **(d)** Same as **(b)** for replicate 10. **(e)** Same as **(c)** for replicate 10. (**f**) Lines plots showing accuracy, precision, sensitivity and F1 score of the reconstructed tissue structures in **(c)** for replicate 9, tissue structure reconstructed with common genes was used as positive reference. (**g**) Same as **(f)** for replicate 10.

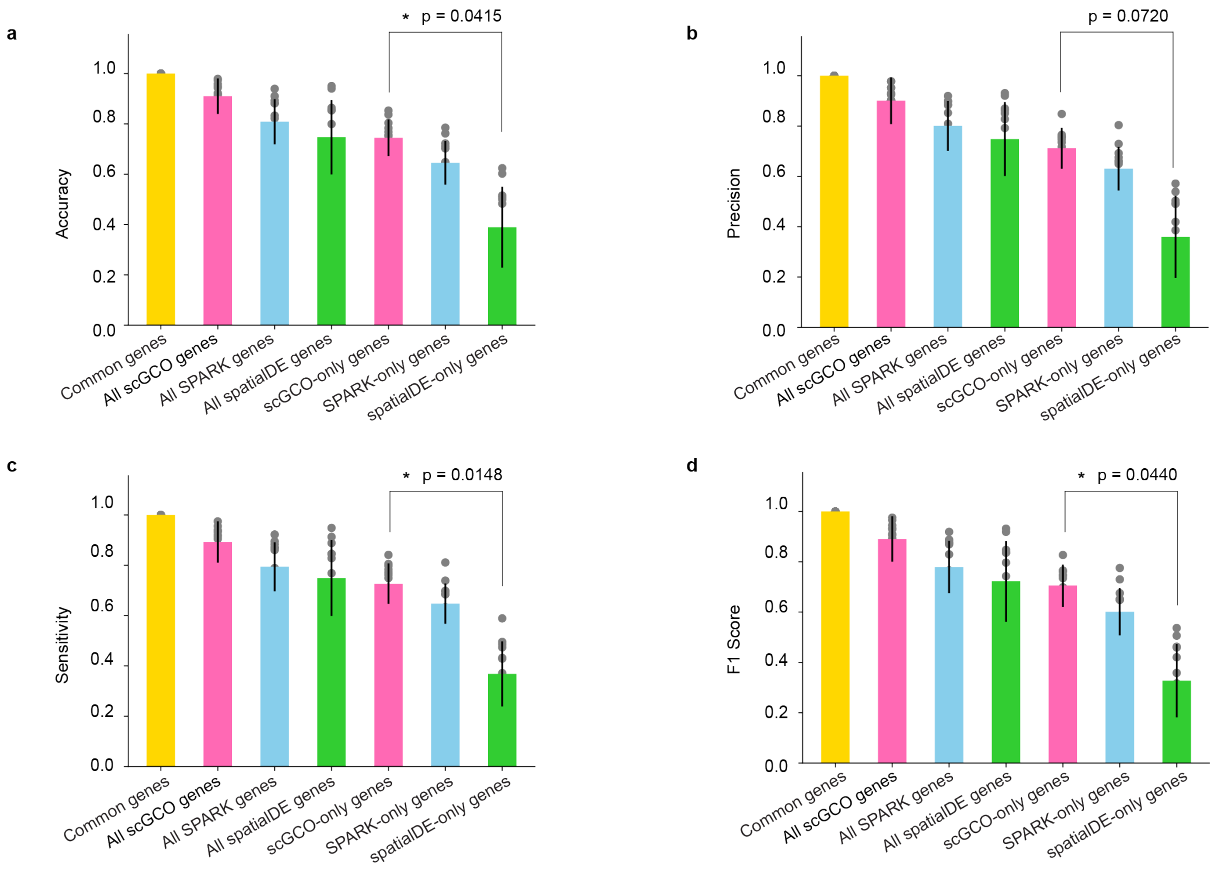

Supplementary Figure 11. Quantifying the performance of identified SV genes to reconstruct MOB tissue structures.

Bar charts showing accuracy (**a**), sensitivity (**b**), precision (**c**) and F1 score (**d**) of reconstructed tissue structures using different sets of SV genes for mouse olfactory bulb dataset (n=12 replicates). MOB tissue architectures reconstructed using SV genes jointly identified by the three methods were used as positive references. Error bars indicate means ± SD. The p-values (p = 0.0415, scGCO-only accuracy vs. spatialDE-only accuracy; p = 0.0720, scGCO-only precision vs. spatialDE-only precision; p = 0.0148, scGCO-only sensitivity vs. spatialDE-only sensitivity; p = 0.0440, scGCO-only F1 score vs. spatialDE-only F1 score) were determined using the two-sided pairwise t-test without multiple-testing correction.

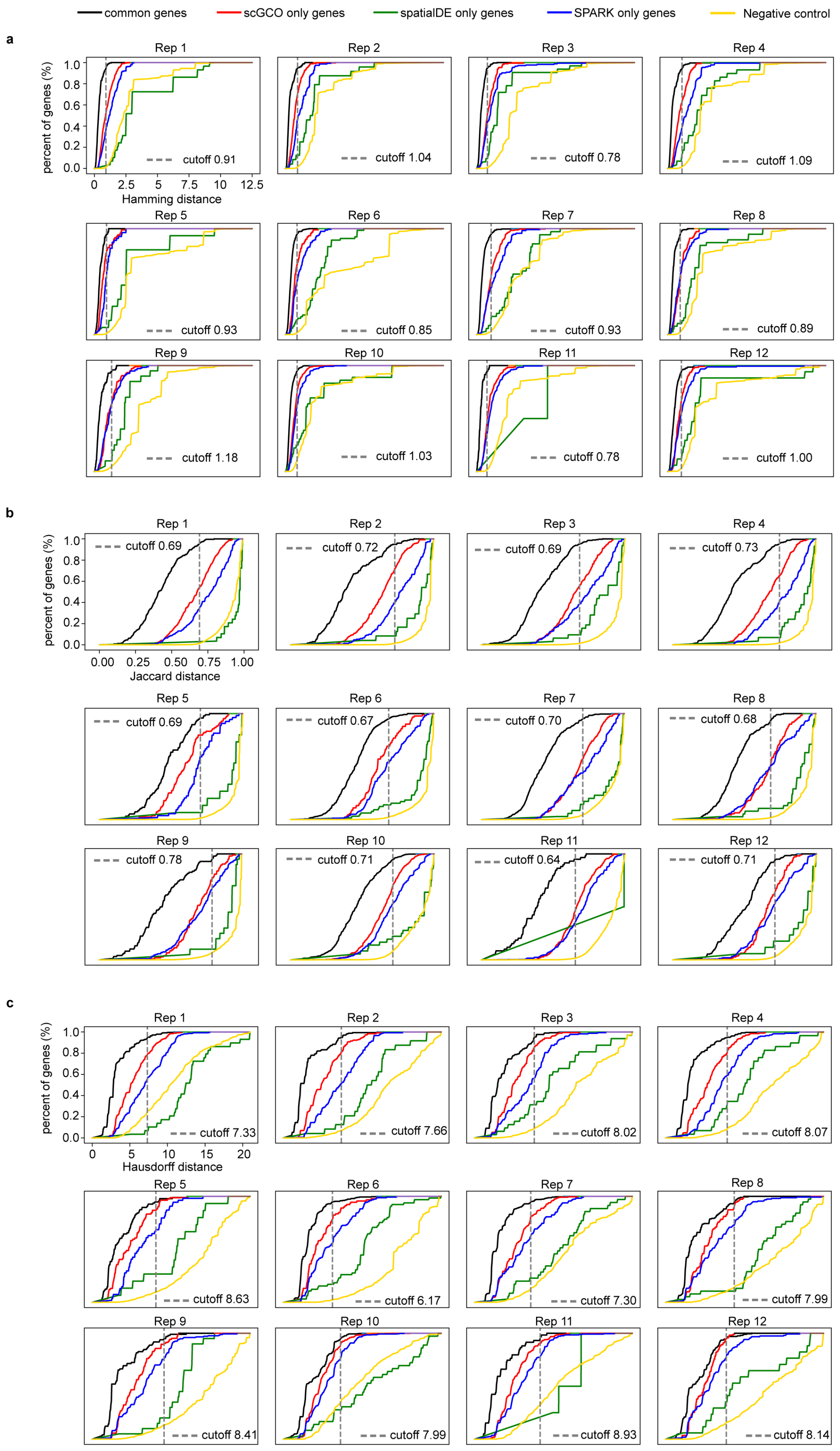

common genes
scGCO only genes
spatialDE only genes
SPARK only genes
Negative control
a
Rep 1
Rep 2
Rep 3
Rep 4
percent of genes (%)
1.0
0.8
0.6
0.4
0.2
0.0
cutoff 0.91
cutoff 1.04
cutoff 0.78
cutoff 1.09
0
2.5
5
7.5
10
12.5
Hamming distance
Rep 5
Rep 6
Rep 7
Rep 8
cutoff 0.93
cutoff 0.85
cutoff 0.93
cutoff 0.89
Rep 9
Rep 10
Rep 11
Rep 12
cutoff 1.18
cutoff 1.03
cutoff 0.78
cutoff 1.00
b
Rep 1
Rep 2
Rep 3
Rep 4
percent of genes (%)
cutoff 0.69
cutoff 0.72
cutoff 0.69
cutoff 0.73
0.00
0.25
0.50
0.75
1.00
Jaccard distance
Rep 5
Rep 6
Rep 7
Rep 8
cutoff 0.69
cutoff 0.67
cutoff 0.70
cutoff 0.68
Rep 9
Rep 10
Rep 11
Rep 12
cutoff 0.78
cutoff 0.71
cutoff 0.64
cutoff 0.71
c
Rep 1
Rep 2
Rep 3
Rep 4
percent of genes (%)
cutoff 7.33
cutoff 7.66
cutoff 8.02
cutoff 8.07
0
5
10
15
20
Hausdorff distance
Rep 5
Rep 6
Rep 7
Rep 8
cutoff 8.63
cutoff 6.17
cutoff 7.30
cutoff 7.99
Rep 9
Rep 10
Rep 11
Rep 12
cutoff 8.41
cutoff 7.99
cutoff 8.93
cutoff 8.14

Supplementary Figure 12. Quantifying similarity of identified SV genes to reference tissue structures.

(a) Line plots showing the cumulative distribution of normalized hamming distance of identified SV genes to reference MOB tissue structures reconstructed with common genes for all 12 replicates. Black lines: SV genes jointly identified by all three methods as positive control; red lines: SV genes uniquely identified by scGCO; green lines: SV genes uniquely identified by spatialDE; blue lines: SV genes uniquely identified by SPARK; gold lines: genes with random spatial gene expression patterns as negative control. The dashed vertical lines indicated estimate distance cutoffs corresponding to 95% of genes jointly identified by all three methods. **(b)** Same as (**a**) using Jaccard distance. **(c)** Same as (**a**) using Hausdorff distance.

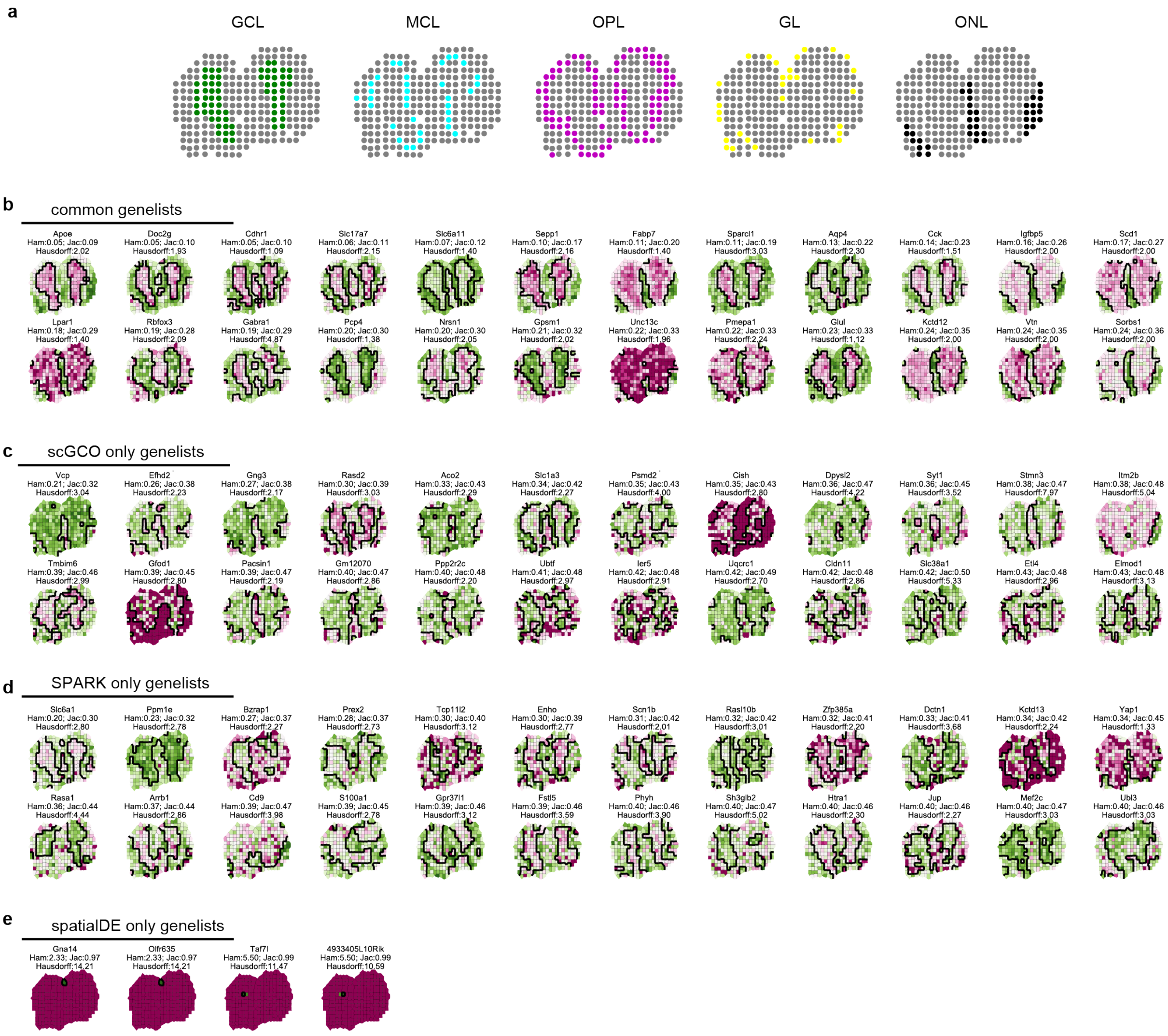

Supplementary Figure 13. Visualizing similarity of identified top ranked SV genes to reference tissue structures.

(a) MOB tissues structures reconstructed using SV genes jointly identified by the three methods as reference for replicate 11. **(b)** Graph cuts showing the top 24 genes with the smallest hamming distance from SV genes jointly reported by all three methods. Turquoise denotes high expression and magenta denotes low expression. **(c)** Same as **(b)** for scGCO-only genes. **(d)** Same as **(b)** for SPARK-only genes. **(e)** Same as **(b)** for spatialDE-only genes (only four genes were uniquely identified by spatialDE).

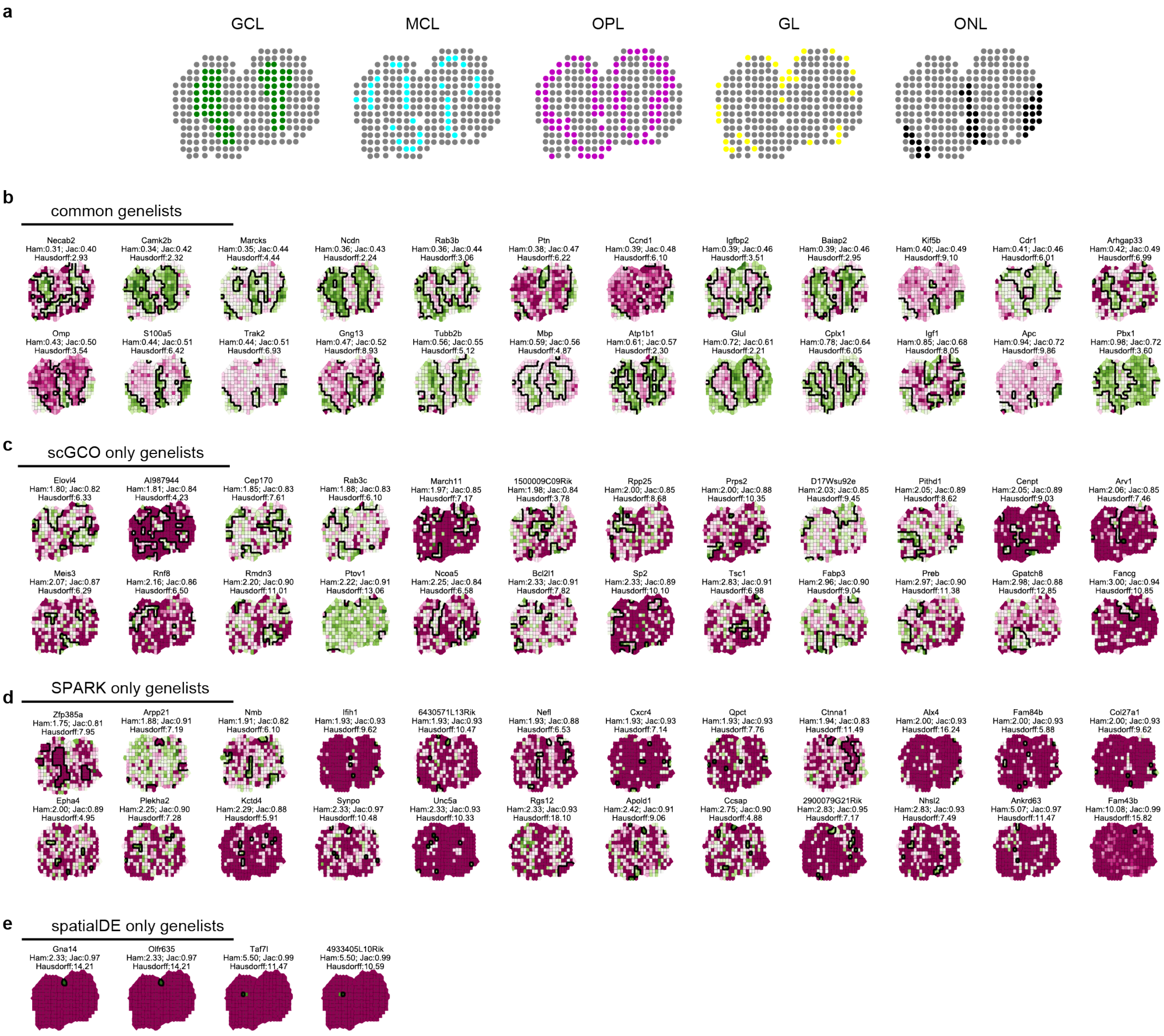

Supplementary Figure 14. Visualizing similarity of bottom ranked SV genes to reference tissue structures.

(a) MOB tissues structures reconstructed using SV genes jointly identified by the three methods as reference for replicate 11. **(b)** Graph cuts showing the bottom 24 genes with the largest hamming distance from SV genes jointly reported by all three methods. Turquoise denotes high expression and magenta denotes low expression. **(c)** Same as **(b)** for scGCO-only genes. **(d)** Same as **(b)** for SPARK-only genes. **(e)** Same as **(b)** for spatialDE-only genes (only four genes were uniquely identified by spatialDE).

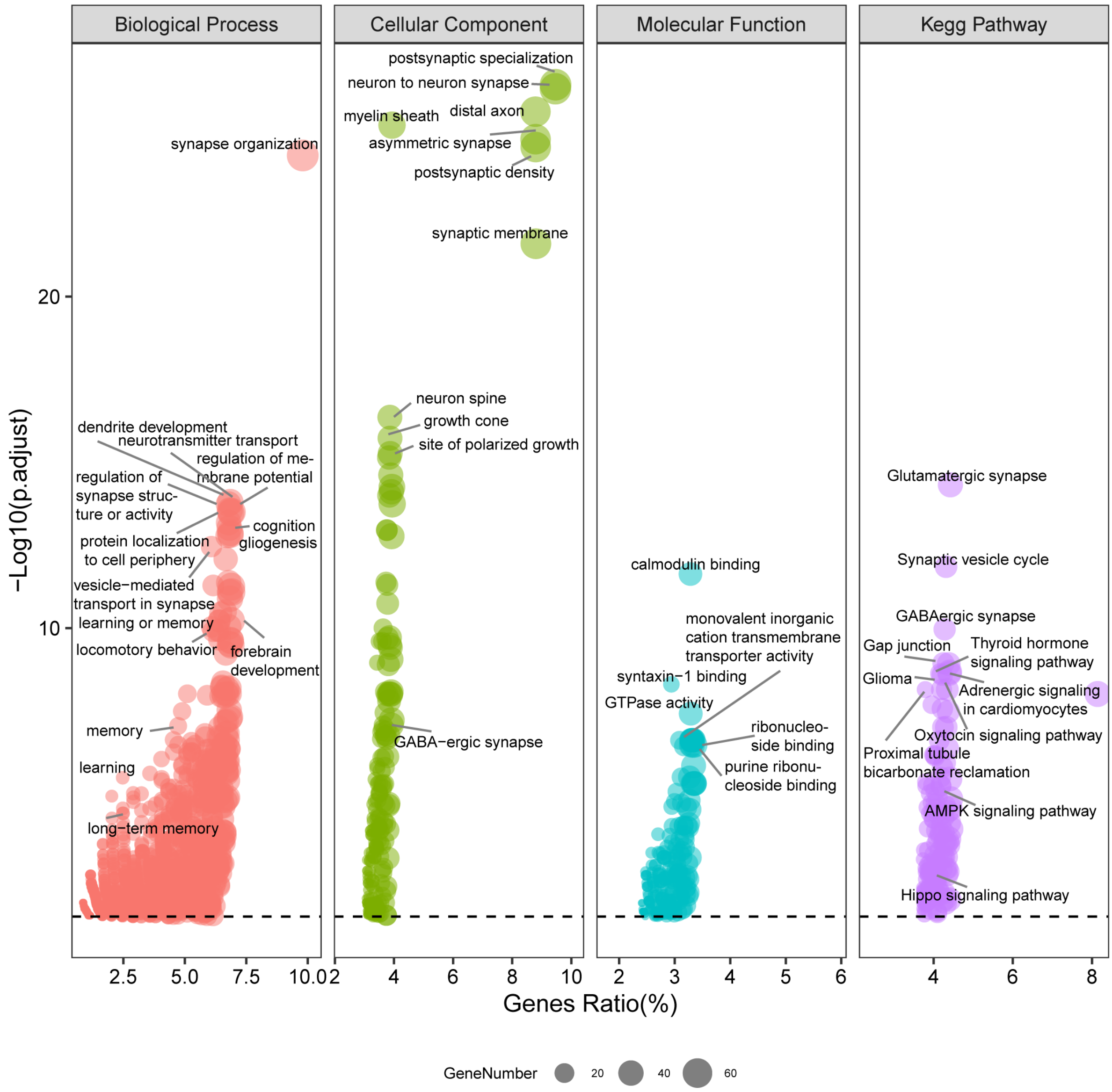

Supplementary Figure 15. SV genes identified by scGCO were enriched with biology functions related to neurons.

Bubble plots showing significantly enriched gene ontologies and KEGG pathways for SV genes identified by scGCO. The horizontal lines correspond to adjusted p-value of 0.05. The adjusted p-values for pathway enrichment analysis were calculated by enrichGO and enrichKEGG functions from clusterProfiler R-package and multiple-testing correction was performed using the Benjamini-Hochberg procedure.

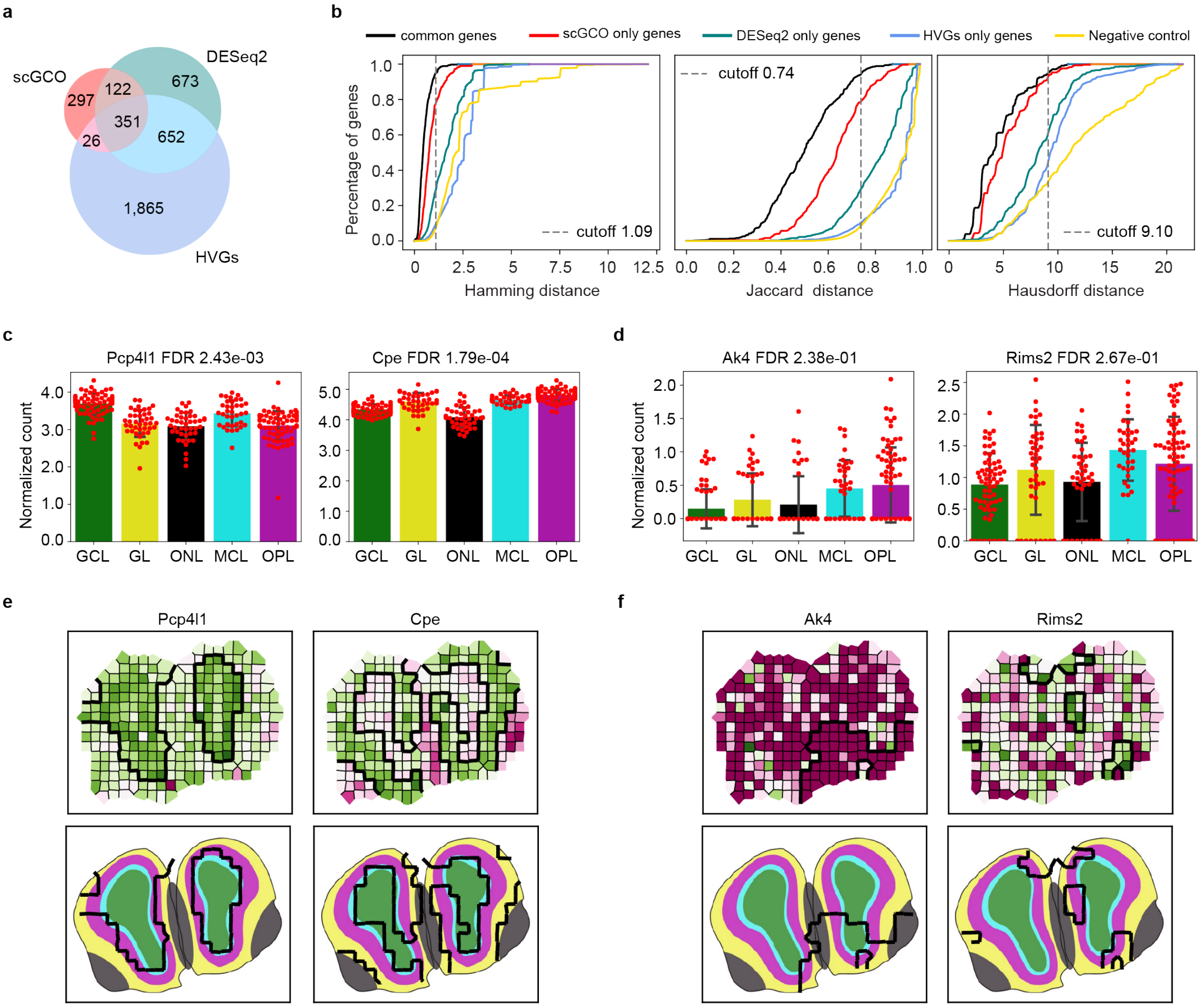

Supplementary Figure 16. Comparing genes identified by scGCO and genes identified by DESeq2.

(**a**) Venn diagram showing the overlap of genes identified by scGCO, HVGs identified with Seurat ignoring spatial context, and genes identified by DESeq2 for replicate 11 of MOB data. DESeq2 genes were identified with FDR < 0.01 and absolute fold change > 1. **(b)** Line plots showing the cumulative distribution of distance similarity of identified SV genes to reference MOB tissue structure for replicate 11 of MOB data. Common genes are the genes jointly identified by scGCO, DESeq2 and HVGs; red lines: SV genes uniquely identified by scGCO; teal lines: SV genes uniquely identified by DESeq2; cornflower blue lines: SV genes uniquely identified by HVGs; gold lines: genes with random spatial expression patterns as negative control. The vertical dashed lines indicate estimate distance cutoffs corresponding to 95% of common genes. **(c)** Bar charts showing the gene expression values in the five layers of MOB tissue for representative scGCO-only genes (GCL=65, GL=42, ONL=43, MCL=36,

and OPL=73). Error bars indicate means ± SD. **(d)** Same as **(c)** for representative DESeq2-only genes (GCL=65, GL=42, ONL=43, MCL=36, and OPL=73). Error bars indicate means ± SD. **(e)** Representative Voronoi diagrams showing graph cuts results (top panels), and associated tissue structure overlays (lower panels) for representative scGCO-only genes. Turquoise denotes high expression and magenta denotes low expression. **(f)** Same as **(e)** for representative DESeq2-only genes.

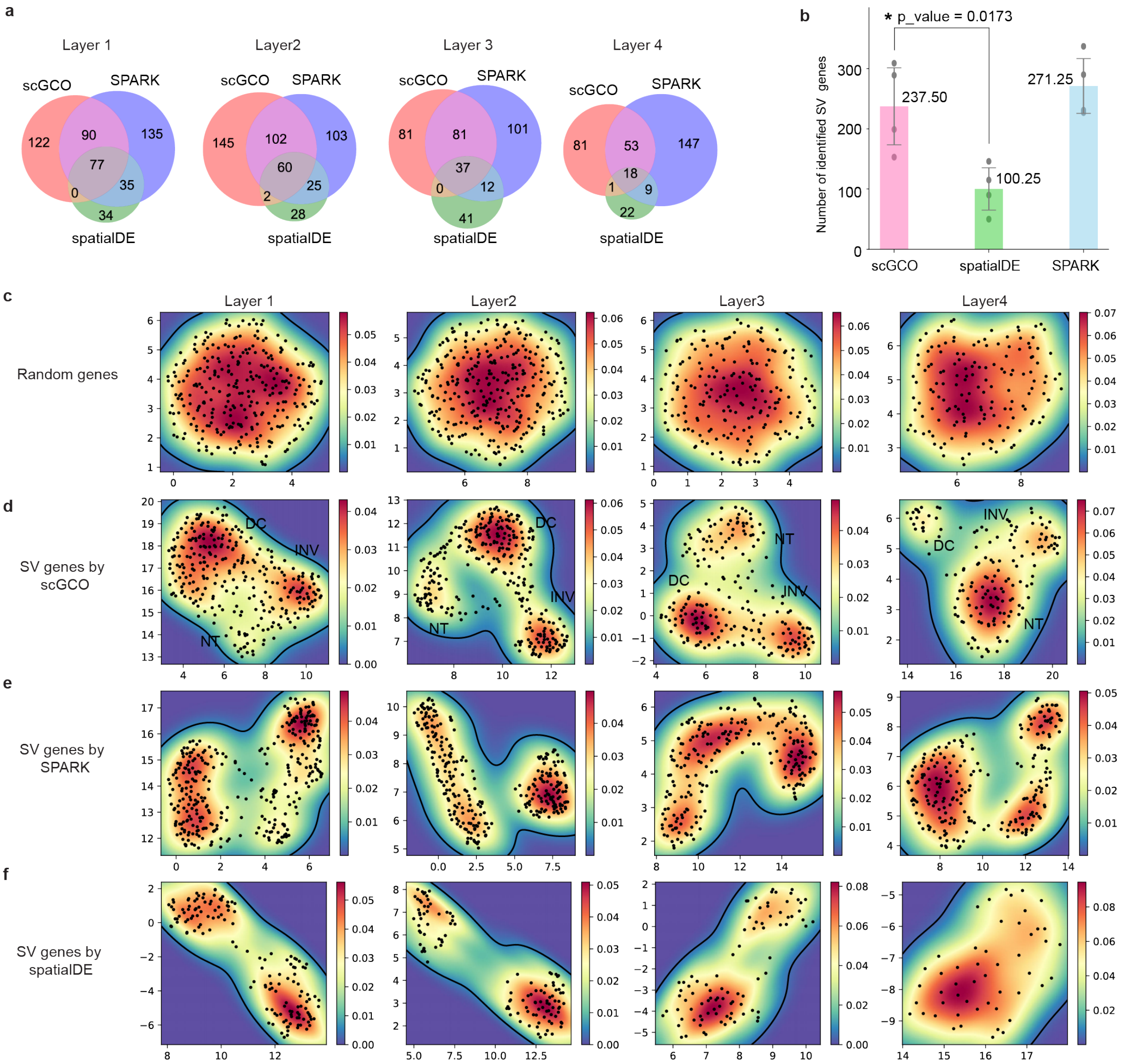

Supplementary Figure 17. Analysis of SV gene identification algorithms using breast cancer biopsies data.

(a) Venn diagram showing the gene set relationship among SV genes identified by scGCO (FDR <0.05), spatialDE (FDR <0.05) and SPARK (FDR <0.05) in breast cancer biopsies. **(b)** Bar plots showing the number of SV genes identified by different methods across all 4 layers, error bars indicate mean ± SD. The p-value (p = 0.0173, scGCO vs. spatialDE) was determined using the two-sided pairwise t-test without multiple-testing correction. **(c)** Kernel density plots of the UMAP projections showing that no clusters were formed among random genes whose expression values were randomly shuffled. **(d)** Kernel density plots of the UMAP projections showing that genes identified by scGCO consistently formed three clusters. **(e)** Kernel density plots of the UMAP projections showing that genes identified by

SPARK consistently formed three clusters. **(f)** Kernel density plots of the UMAP projections showing that genes identified by spatialDE didn't consistently form three clusters.

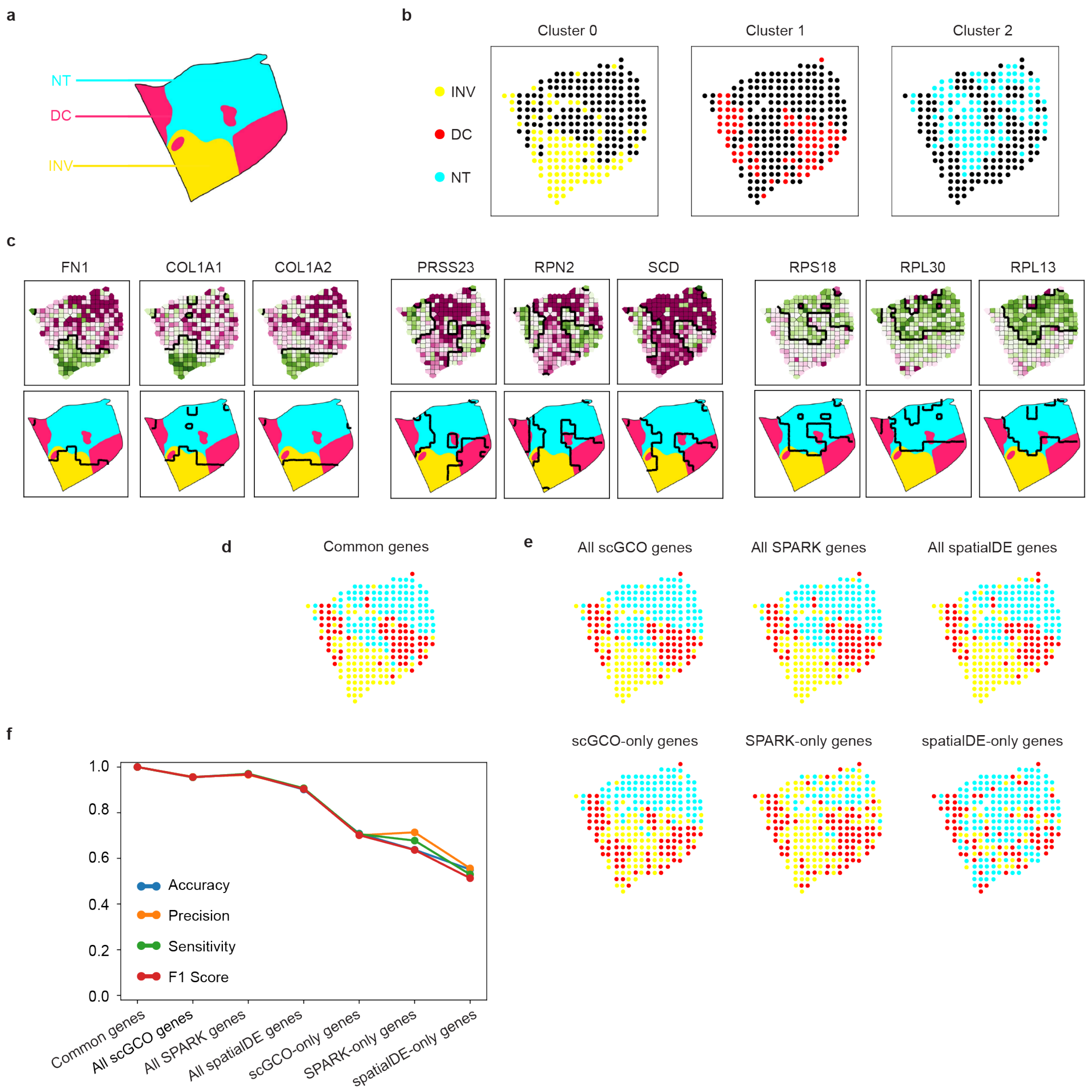

Supplementary Figure 18. SV genes identified by scGCO reconstruct breast cancer tissue structures.

(**a**) Annotation of the breast cancer tissue architecture showing three regions: INV, DC and NT for layer 1. INV: Invasive Ductal Cancer; DC: Ductal Cancer; NT: Normal Tissue. **(b)** Tissue domains resolved with each cluster of genes identified by scGCO for layer 1. **(c)** Representative graph cuts and tissue structure overlays for SV genes identified by scGCO (layer 1). Voronoi diagrams showing graph cuts results (top panels), and associated tissue structure overlays (lower panels). Turquoise denotes high expression and magenta denotes low expression. **(d)** Reconstructed tissue architecture for layer 1 with SV genes jointly

identified by all three methods. **(e)** Breast cancer tissue structures reconstructed using all scGCO genes (upper column 1), all SPARK genes (upper column 2), all spatialDE genes (upper column 3), scGCO-only genes (lower column 1), SPARK-only genes (lower column 2), and spatialDE-only genes (lower column 3) for layer 1. **(f)** Lines plots showing accuracy, precision, sensitivity and F1 score of the reconstructed cancer tissues in **(e)**, tissue structure reconstructed with common genes was used as reference.

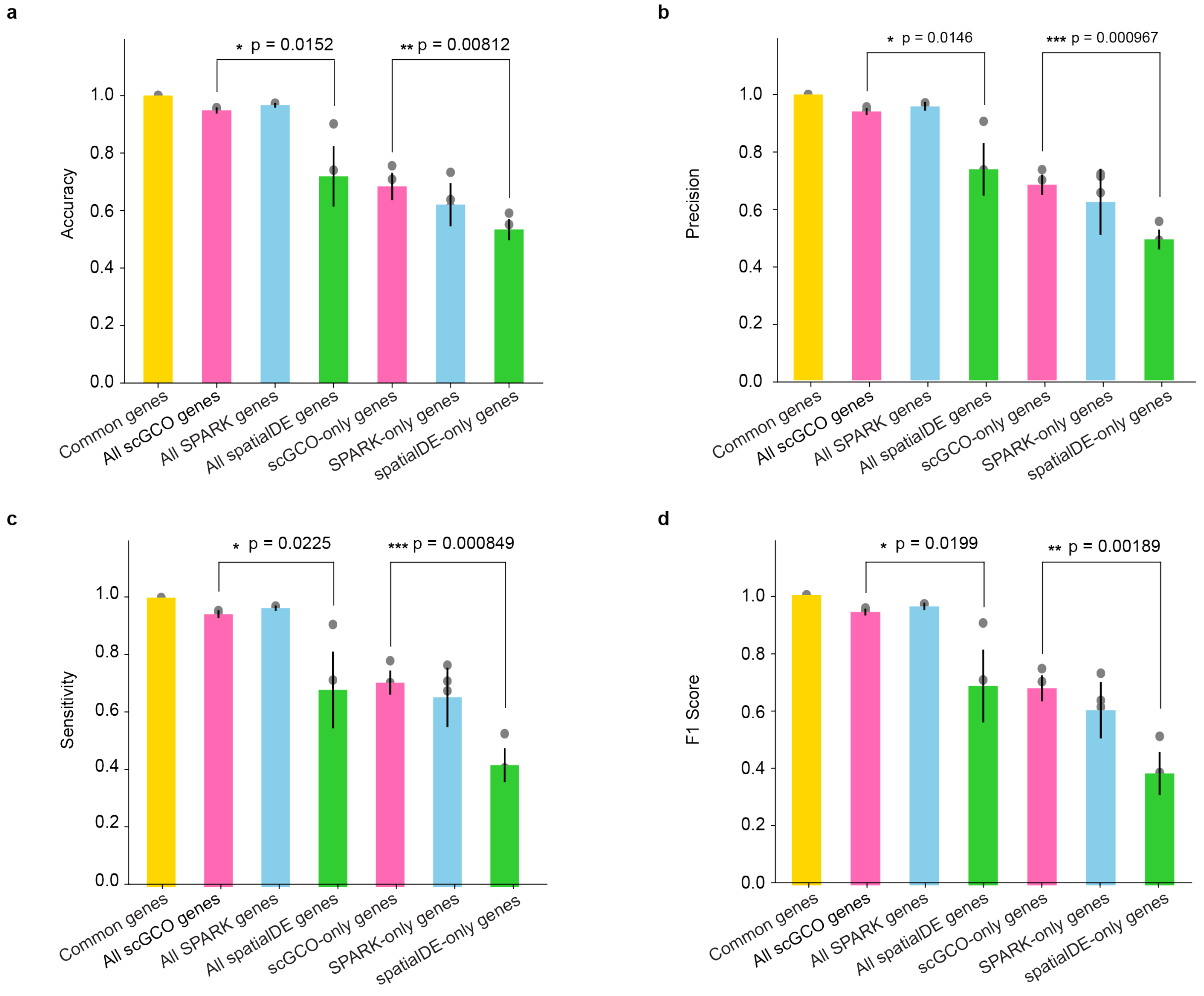

Supplementary Figure 19. Quantifying the performance of identified SV genes to resolve Breast Cancer tissue structures.

Bar plots showing the Accuracy **(a)**, Precision **(b)**, Sensitivity **(c)** and F1 score **(d)** of reconstructed tissue structures using different sets of SV genes for all breast cancer data (n= 4) layers, tissue structure reconstructed with common genes (SV genes jointly identified by all three methods) was used as reference. Error bars indicate means ± SD. The p-values (p = 0.0152, all scGCO accuracy vs. all spatialDE accuracy; p = 0.00812, scGCO-only accuracy vs. spatialDE-only accuracy; p = 0.0146, all scGCO precision vs. all spatialDE precision; p = 0.000967, scGCO-only precision vs. spatialDE-only precision; p = 0.0225, all scGCO sensitivity vs. all spatialDE sensitivity; p = 0.000849, scGCO-only sensitivity vs. spatialDE-only sensitivity; p = 0.0199, all scGCO F1 score vs. all spatialDE F1 score; p = 0.00189, scGCO-only F1 score vs. spatialDE-only F1 score) were determined using the two-sided pairwise t-test without multiple-testing correction.

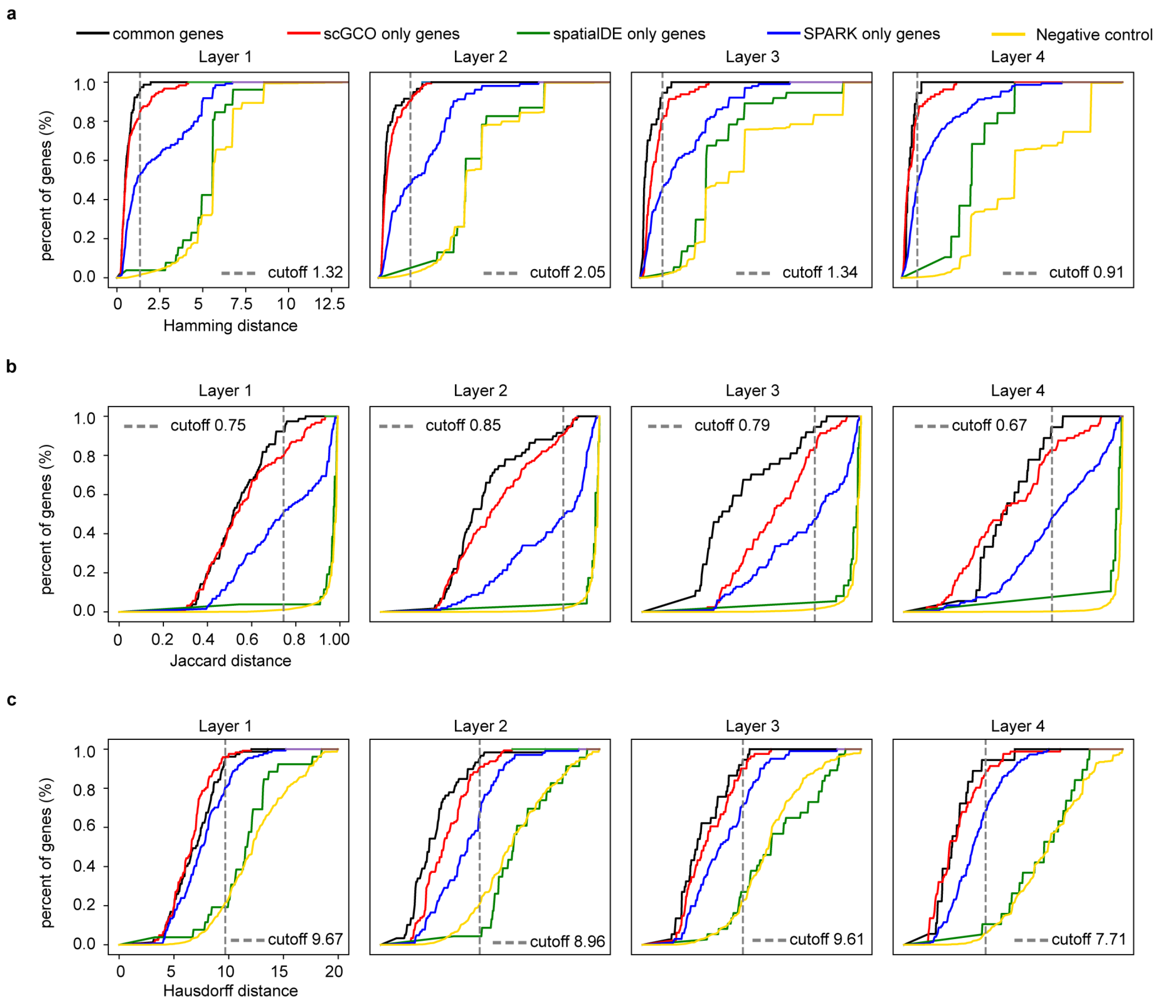

Supplementary Figure 20. Quantifying similarity of identified SV genes to reference cancer tissue structures.

(a) Line plots showing the cumulative distribution of normalized hamming distance of identified SV genes to reference BC tissue structures reconstructed using common genes. Black lines: SV genes jointly identified by all three methods; red lines: SV genes uniquely identified by scGCO; green lines: SV genes uniquely identified by spatialDE; blue lines: SV genes uniquely identified by SPARK; gold lines: genes with random spatial expression patterns as negative control. The dashed vertical lines indicate estimate distance cutoffs corresponding to 95% of common genes. **(b)** Same as (**a**) using Jaccard distance. **(c)** Same as (**a**) using Hausdorff distance.

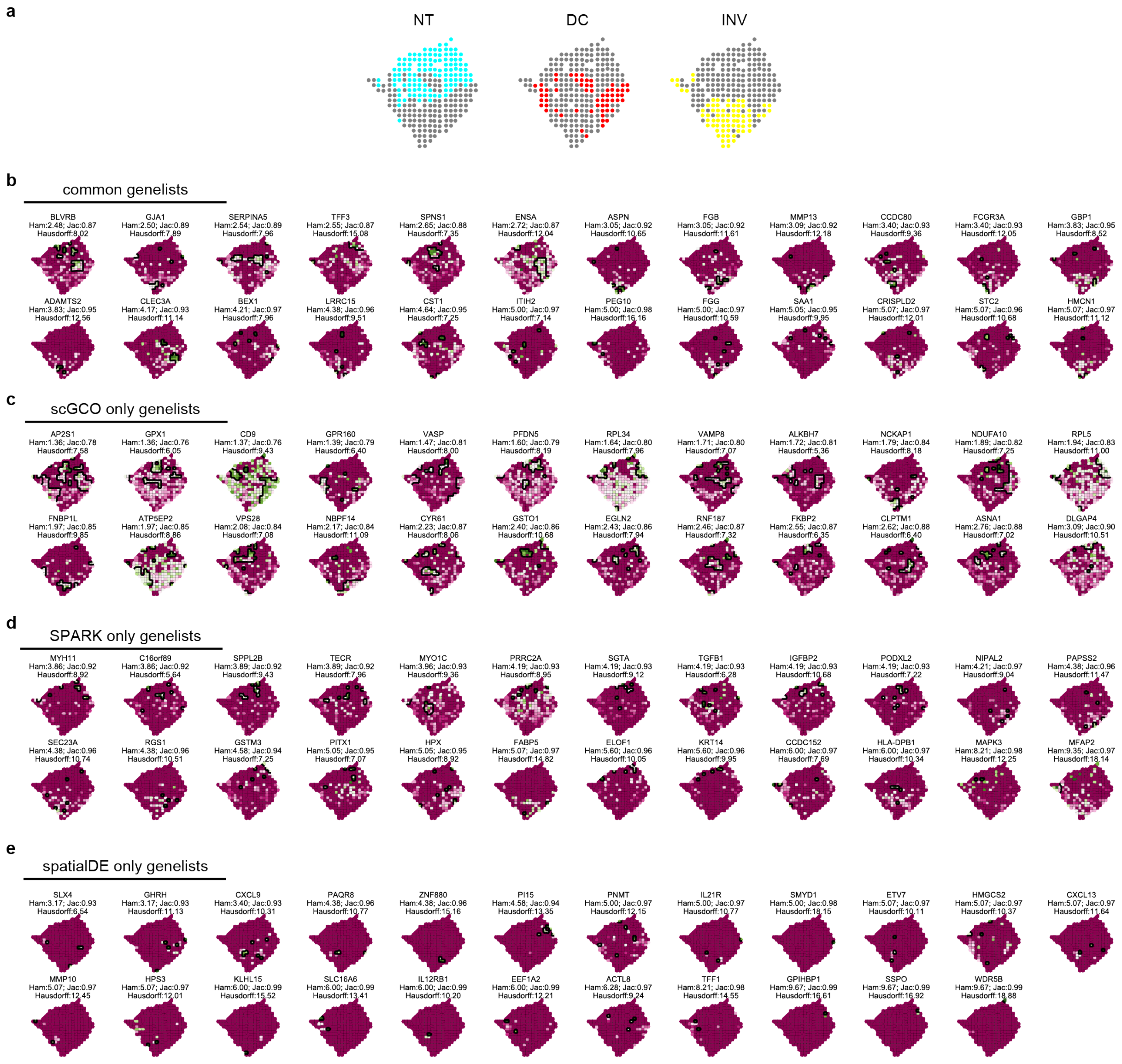

Supplementary Figure 21. Visualizing similarity of identified SV genes to reference breast cancer tissue structures.

(a) Reference cancer tissues structures reconstructed with common genes for layer 2. **(b)** Graph cuts showing bottom 24 genes with the largest hamming distance for common genes. Turquoise denotes high expression and magenta denotes low expression. **(c)** Same as **(b)** for scGCO-only genes. **(d)** Same as **(b)** for SPARK-only genes. **(e)** Same as **(b)** for spatialDE-only genes.

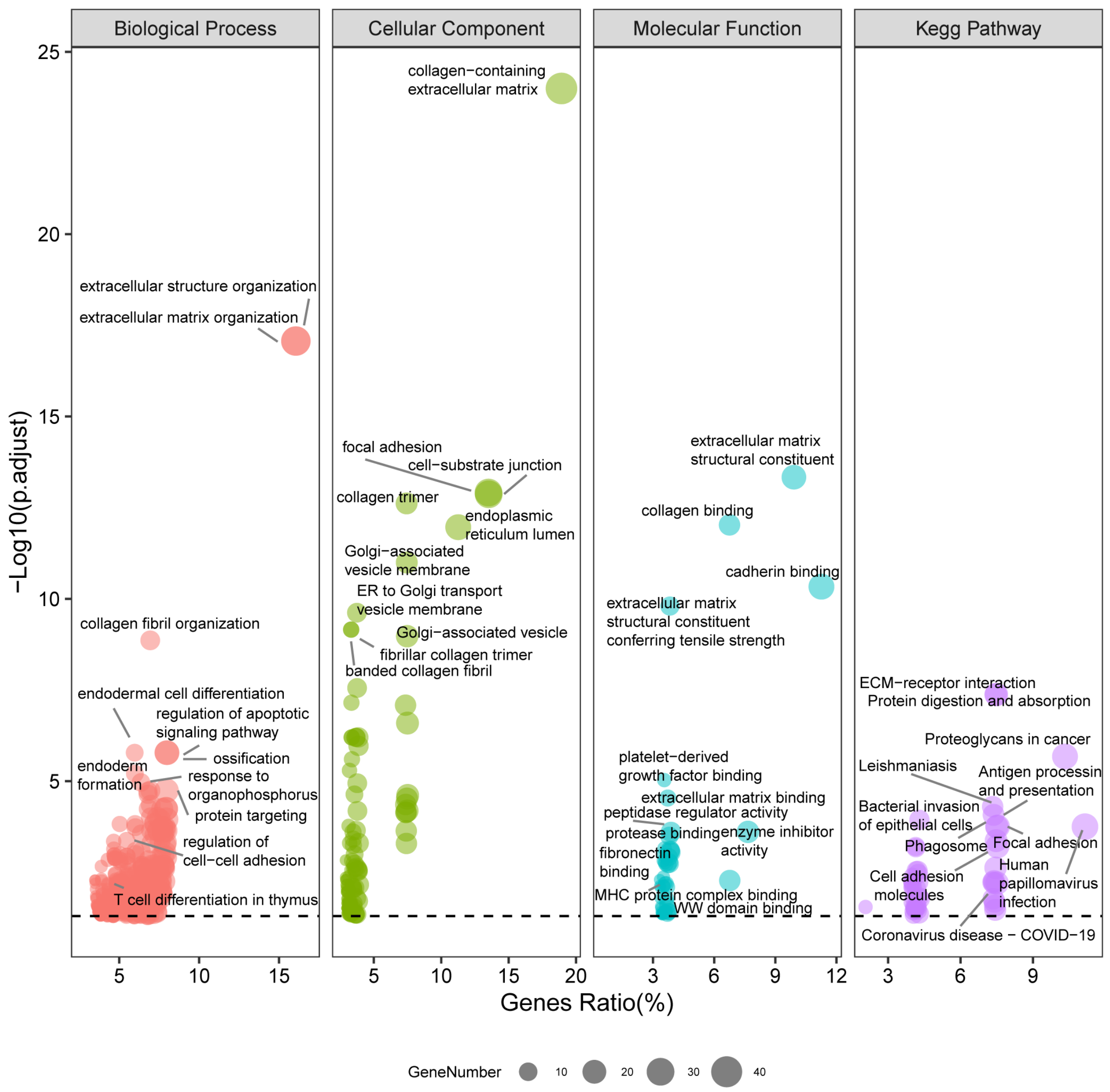

Supplementary Figure 22. SV genes identified by scGCO are enriched with cancer-related function.

Bubble plots showing significantly enriched gene ontologies and KEGG pathways for SV genes identified by scGCO from layer 2. The horizontal lines correspond to adjusted p-value of 0.05. The adjusted p-values for pathway enrichment analysis were calculated by enrichGO and enrichKEGG functions from clusterProfiler R-package and multiple-testing correction was performed using the Benjamini-Hochberg procedure.

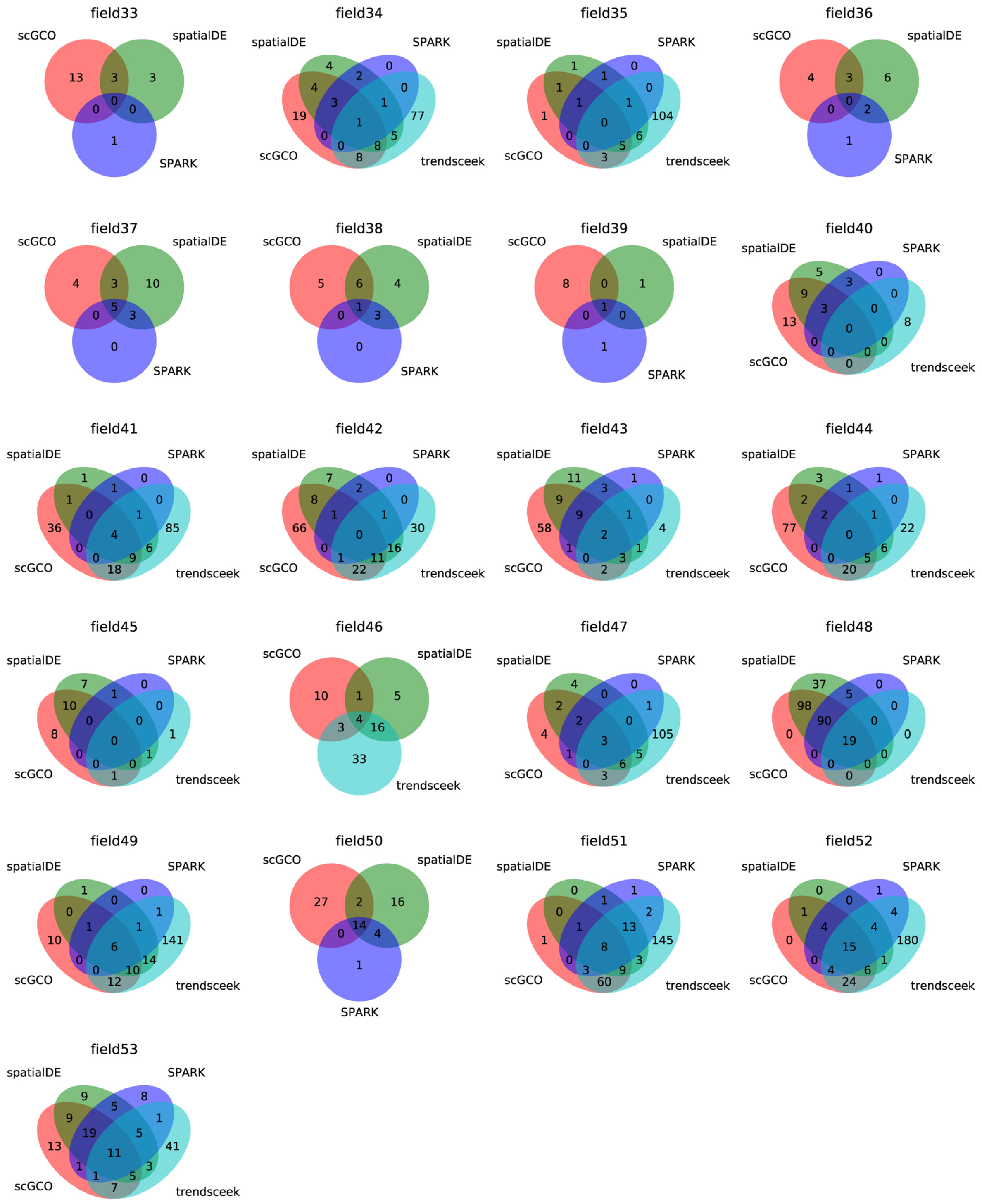

Supplementary Figure 23. Identifying SV genes in mouse hippocampus seqFISH data.

Venn diagram showing the gene set relationship among SV genes identified by scGCO, spatialDE, SPARK and trendSceek from mouse hippocampus seqFISH data (FDR < 0.05).

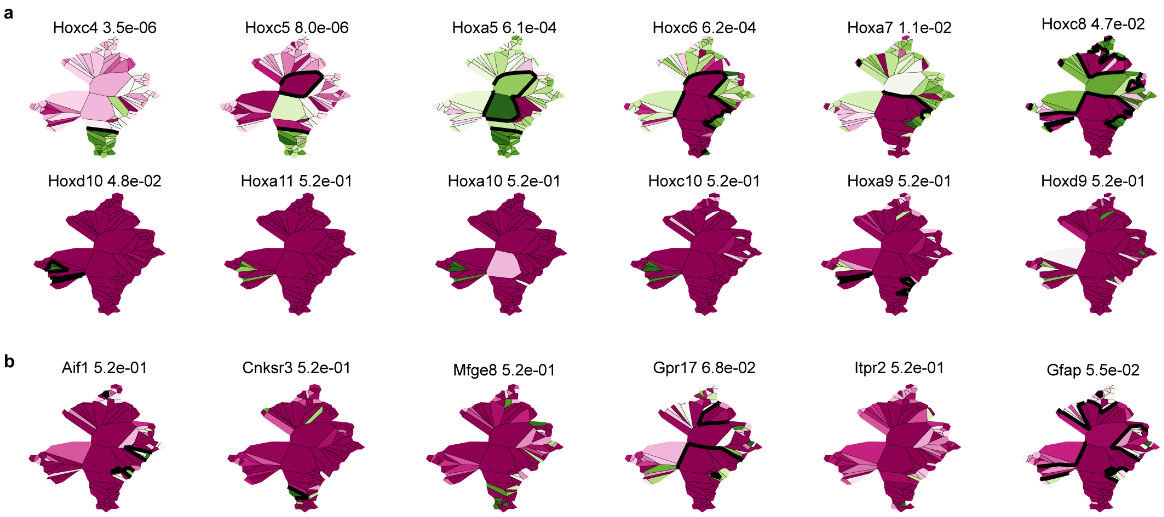

Supplementary Figure 24. Identifying SV genes in mouse neurons tissues with LCM-seq technology.

(a) Spatial patterns for twelve Hox genes. **(b)** same as **(a)** for six non-spatial expression genes. Turquoise denotes high expression and magenta denotes low expression. Thicker black lines denote graph cuts boundaries. The adjusted p-values were calculated by scGCO and multiple-testing correction was performed using the Benjamini–Hochberg procedure.

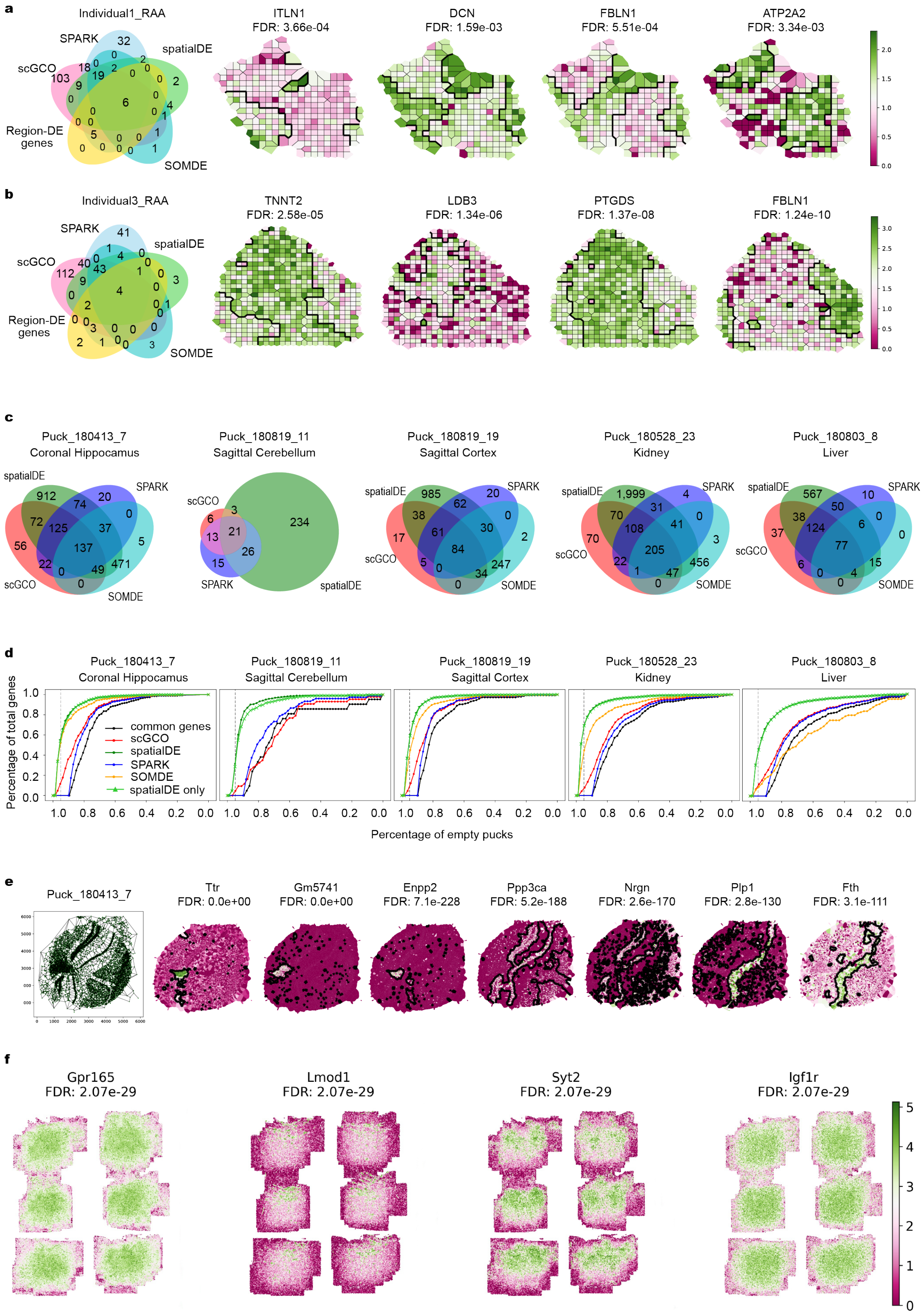
a
Individual1_RAA
SPARK
spatialDE
scGCO
Region-DE genes
SOMDE
ITLN1
FDR: 3.66e-04
DCN
FDR: 1.59e-03
FBLN1
FDR: 5.51e-04
ATP2A2
FDR: 3.34e-03
b
Individual3_RAA
TNNT2
FDR: 2.58e-05
LDB3
FDR: 1.34e-06
PTGDS
FDR: 1.37e-08
FBLN1
FDR: 1.24e-10
c
Puck_180413_7
Coronal Hippocampus
Puck_180819_11
Sagittal Cerebellum
Puck_180819_19
Sagittal Cortex
Puck_180528_23
Kidney
Puck_180803_8
Liver
d
Percentage of total genes
common genes
scGCO
spatialDE
SPARK
SOMDE
spatialDE only
Percentage of empty pucks
e
Puck_180413_7
Ttr
FDR: 0.0e+00
Gm5741
FDR: 0.0e+00
Enpp2
FDR: 7.1e-228
Ppp3ca
FDR: 5.2e-188
Nrgn
FDR: 2.6e-170
Plp1
FDR: 2.8e-130
Fth
FDR: 3.1e-111
f
Gpr165
FDR: 2.07e-29
Lmod1
FDR: 2.07e-29
Syt2
FDR: 2.07e-29
Igf1r
FDR: 2.07e-29

Supplementary Figure 25. Identifying SV genes with three benchmark data sets.

(a) Venn diagram showing the gene set relationship among Region-DE genes and SV genes identified by scGCO, spatialDE, SPARK and SOMDE from right atrial appendage (RAA) of individual 1with ST sequencing (Spatial Transcriptomic) (FDR < 0.05). Spatial patterns for representative SV genes were shown. Turquoise denotes high expression and magenta denotes low expression. Thicker black lines denote graph cuts boundaries. The adjusted p-values were calculated by scGCO and multiple-testing correction was performed using the Benjamini–Hochberg procedure. **(b)** same as **(a)** for individual 3. **(c)** Venn diagram showing the gene set relationship among SV genes identified by scGCO, spatialDE, SPARK and SOMDE from five complex tissue with slide-seq technology (n=5) (FDR < 0.05). **(d)** Line plots showing the cumulative distribution of percentage of empty pucks for SV genes identified by different methods from the five complex tissues in **(c)**. Black lines: common genes jointly identified by scGCO, spatialDE, SPARK, and SOMDE; red lines: SV genes identified by scGCO; green lines: SV genes identified by spatialDE; blue lines: SV genes identified by SPARK; orange lines: SV genes identified by SOMDE; light green lines with triangular symbol: SV genes uniquely identified by spatialDE. The dashed vertical line represents the 95 percentage of empty pucks. **(e)** Representative Voronoi diagrams and spatial patterns of SV genes for coronal hippocamus in **(c)**. The adjusted p-values were calculated by scGCO and multiple-testing correction was performed using the Benjamini–Hochberg procedure. **(f)** Spatial patterns for representative SV genes identified by scGCO from mouse hypothalamus with MERFISH technology. Turquoise denotes high expression and magenta denotes low expression. The adjusted p-values were calculated by scGCO and multiple-testing correction was performed using the Benjamini–Hochberg procedure.
