# Supplementary material for: Identification of spatially variable genes with graph cuts
Source: Nat Commun. 2022 Sep 19;13:5488. doi: 10.1038/s41467-022-33182-3 (PMC9485129; doi:10.1038/s41467-022-33182-3)
Supplement: Supplementary file 2 — Reporting Summary [file 41467_2022_33182_MOESM2_ESM.pdf]

Corresponding author(s): Peng Wang
Last updated by author(s): Sep 3, 2022

Reporting Summary

Nature Portfolio wishes to improve the reproducibility of the work that we publish. This form provides structure for consistency and transparency in reporting. For further information on Nature Portfolio policies, see our Editorial Policies and the Editorial Policy Checklist.

Statistics

For all statistical analyses, confirm that the following items are present in the figure legend, table legend, main text, or Methods section.

n/a	Confirmed	
☐	☒	The exact sample size (n) for each experimental group/condition, given as a discrete number and unit of measurement
☒	☐	A statement on whether measurements were taken from distinct samples or whether the same sample was measured repeatedly
☐	☒	The statistical test(s) used AND whether they are one- or two-sided<br>*Only common tests should be described solely by name; describe more complex techniques in the Methods section.*
☒	☐	A description of all covariates tested
☐	☒	A description of any assumptions or corrections, such as tests of normality and adjustment for multiple comparisons
☐	☒	A full description of the statistical parameters including central tendency (e.g. means) or other basic estimates (e.g. regression coefficient) AND variation (e.g. standard deviation) or associated estimates of uncertainty (e.g. confidence intervals)
☐	☒	For null hypothesis testing, the test statistic (e.g. F, t, r) with confidence intervals, effect sizes, degrees of freedom and P value noted<br>*Give P values as exact values whenever suitable.*
☒	☐	For Bayesian analysis, information on the choice of priors and Markov chain Monte Carlo settings
☒	☐	For hierarchical and complex designs, identification of the appropriate level for tests and full reporting of outcomes
☒	☐	Estimates of effect sizes (e.g. Cohen's d, Pearson's r), indicating how they were calculated

Our web collection on statistics for biologists contains articles on many of the points above.

Software and code

Policy information about availability of computer code

Data collection	No softwares were involved in data collection. All data used in this study was downloaded from websites described in the data availability statement below.
Data analysis	To systematically evaluate the performance of scGCO against published algorithms, we evaluated spatialDE (version 1.1.1), trendSceek (version 1.0.0), SPARK (version 1.0.2), and SOMDE (version 0.1.8). For spatialDE and SPARK, we downloaded the scripts provided by the authors from their GitHub website and executed the scripts without modification. For SOMDE, we installed the python package. For trendSceek, we implemented R scripts according to the methods descripted in trendSceek's original paper. The trendSceek's scripts and the scripts to run scGCO are provided in the tutorial files in scGCO's GitHub repository. Pairwise differential expression analyses were performed between all possible pairs of groups with DESeq2 using default parameters (version 1.22.2). HVGs were identified using Seurat's FindVariableGenes function (version 2.3.4). The gene ontology and pathway enrichment analyses were performed using the enrichGO and enrichKEGG functions from clusterProfiler R-package (v 3.16.0). An open-source implementation of scGCO is available at GitHub (https://github.com/WangPeng-Lab/scGCO).

For manuscripts utilizing custom algorithms or software that are central to the research but not yet described in published literature, software must be made available to editors and reviewers. We strongly encourage code deposition in a community repository (e.g. GitHub). See the Nature Portfolio guidelines for submitting code & software for further information.

Data

Policy information about availability of data

All manuscripts must include a data availability statement. This statement should provide the following information, where applicable:

- Accession codes, unique identifiers, or web links for publicly available datasets
- A description of any restrictions on data availability
- For clinical datasets or third party data, please ensure that the statement adheres to our policy

We downloaded the spatial transcriptomics data reported by Ståhl et al. from the Spatial Transcriptomics Research website (https://www.spatialresearch.org/resources-published-datasets/doi-10-1126science-aaf2403/). We used all 12 replicates for the mouse olfactory bulb, and all four layers for the breast cancer data. For mouse hippocampus seqFISH data, we downloaded the data from https://ars.els-cdn.com/content/image/1-s2.0-S0896627316307024-mmc6.xlsx. We used all 21 fields provided by the authors for analysis. The MERFISH data was downloaded from the Zhuang lab website (http://zhuang.harvard.edu/merfish.html). We used "Replicate 6" similar to spatialDE8, as these had the largest number of cells and highest confluency. The LCM-seq data was downloaded from Gene Expression Omnibus (GEO) of the National Center for Biotechnology Information under the accession number GSE60402 [https://www.ncbi.nlm.nih.gov/geo/query/acc.cgi?acc=GSE60402] and GSE76514 [https://www.ncbi.nlm.nih.gov/geo/query/acc.cgi?acc=GSE76514]. The ST sequencing and slide-seq data used in this study have been available in the SpatialDB database with website: (http://www.spatialomics.org/SpatialDB/).

Human research participants

Policy information about studies involving human research participants and Sex and Gender in Research.

Reporting on sex and gender	No human subject was involved in this study.
Population characteristics	No human subject was involved in this study.
Recruitment	No human subject was involved in this study.
Ethics oversight	No human subject was involved in this study.

Note that full information on the approval of the study protocol must also be provided in the manuscript.

Field-specific reporting

Please select the one below that is the best fit for your research. If you are not sure, read the appropriate sections before making your selection.

[x] Life sciences [ ] Behavioural & social sciences [ ] Ecological, evolutionary & environmental sciences

For a reference copy of the document with all sections, see nature.com/documents/nr-reporting-summary-flat.pdf

Life sciences study design

All studies must disclose on these points even when the disclosure is negative.

Sample size	For the simulated data set, we simulated 10 samples for each condition following the standard practice in machine learning community. For public data sets, sample sizes were determined by the authors generating these data.
Data exclusions	No data was excluded from analysis.
Replication	For the simulated data set, we simulated 10 samples for each condition. And calculated standard deviation to verify the reproducibility. For public data sets, reproducibilities were determined by the authors generating these data.
Randomization	Randomization was not relevant to our study because we only used publicly available data. And for simulated data no life science study is involved.
Blinding	Blinding was not relevant to our study because we only used publicly available data. And for simulated data no life science study is involved.

Reporting for specific materials, systems and methods

We require information from authors about some types of materials, experimental systems and methods used in many studies. Here, indicate whether each material, system or method listed is relevant to your study. If you are not sure if a list item applies to your research, read the appropriate section before selecting a response.

Materials & experimental systems

n/a	Involved in the study
☒	☐ Antibodies
☒	☐ Eukaryotic cell lines
☒	☐ Palaeontology and archaeology
☒	☐ Animals and other organisms
☒	☐ Clinical data
☒	☐ Dual use research of concern

Methods

n/a	Involved in the study
☒	☐ ChIP-seq
☒	☐ Flow cytometry
☒	☐ MRI-based neuroimaging
